# *IbMYB11* Involved in Drought Stress Responses Through Association with Flavonoid Metabolism in Sweet Potato

**DOI:** 10.3390/plants15203119

**Published:** 2026-10-12

**Authors:** Zhengqian Luo, Ruitao Liu, Linli Xu, Yuehua Yu, Zifeng Lu, Zhouwen He, Yunxiao Wang, Xian Wang, Li Xiang, Yuhui Xu, Enliang Liu

**Affiliations:** 1 Xinjiang Academy of Agricultural Science, Urumqi 830091, China; 2 College of Agronomy and Biotechnology, China Agriculture University, Beijing 100193, China; 3 College of Agronomy, Xinjiang Agricultural University, Urumqi 830052, China

**Keywords:** sweet potato, drought stress, multiomics, transcription factor, flavonoid metabolism, gene function

## Abstract

Sweet potato (*Ipomoea batatas* (L.) Lam.) is a high-yielding crop that serves as both a food source and a raw material for industrial processing. Drought stress severely impairs sweet potato yield and quality. A MYB-type transcription factor, *IbMYB11*, was previously identified as a differentially expressed gene induced by drought stress from our previous comparative transcriptome analysis. In this study, we characterized its potential roles in drought tolerance and flavonoid metabolism in sweet potato. Subcellular localization analysis revealed that IbMYB11 localizes to the nucleus. Overexpression of *IbMYB11* enhanced SOD and POD activities in transgenic sweet potato plants under drought stress, whereas these activities were significantly reduced in *IbMYB11*-RNAi plants, suggesting that *IbMYB11* may confer drought tolerance by improving the capacity to scavenge reactive oxygen species. Transcriptome and metabolome analyses of *IbMYB11*-OE, *IbMYB11*-RNAi, and wild-type plants under simulated drought stress further revealed that the differentially expressed genes (DEGs) from all comparison groups were enriched in the flavonoid biosynthesis pathway, and nine flavonoid metabolites were identified among the differentially accumulated metabolites. Furthermore, differentially expressed TFs and flavonoid metabolites were jointly subjected to WGCNA, yielding 12 hub genes and modules significantly associated with flavonoid metabolites. Collectively, this study demonstrates that *IbMYB11* improved drought tolerance, likely in part via its association with flavonoid metabolism, and providing valuable genetic resources for molecular design breeding of drought-resistant sweet potato.

## 1. Introduction

Sweet potato (*Ipomoea batatas* (L.) Lam.; 2n = 6x = 90), belonging to the family Convolvulaceae and genus *Ipomoea*, represents a globally significant food crop, feed resource, and industrial raw material owing to its broad adaptability, high yield potential, and tolerance to marginal soils specially in Asia and Africa [1]. The storage roots of sweet potato are rich in starch, dietary fiber, vitamins, and minerals. Furthermore, specific cultivars accumulate substantial quantities of bioactive compounds, including β-carotene, which exhibit remarkable antioxidant, anti-inflammatory, and anti-carcinogenic properties, rendering sweet potato not merely a staple food but also a functional health food [2,3]. Beyond its nutritional value, sweet potato plays a pivotal role in industrial production; its high starch content enables the manufacture of bioethanol, starch-based sugars, citric acid, and biodegradable plastics [4]. However, despite its considerable environmental resilience, the growth, development, and tuberous root yield of sweet potato remain severely constrained by multiple abiotic stresses, among which drought represents one of the most pervasive and detrimental factors. Global climate change has led to an escalating frequency and intensity of extreme drought events, posing unprecedented threats to agricultural production. Throughout the entire growth cycle of sweet potato, sustained or intermittent drought stress critically impairs photosynthesis, biomass accumulation, and nutrient partitioning, resulting in retarded plant growth, reduced leaf area, decreased biomass, and ultimately significant reductions in root yield and quality [5]. Consequently, elucidating the molecular regulatory networks underlying sweet potato responses to drought stress, identifying and exploiting superior drought-resistance genetic resources, and breeding new high-yielding, stable-yielding, drought-tolerant sweet potato varieties carry profound theoretical significance urgent practical importance for safeguarding global food security, promoting sustainable agricultural development, and addressing the challenges posed by climate change.

Over the past two decades, systematic research strategies have substantially advanced our understanding of the molecular mechanisms related to drought stress. These studies have demonstrated that plants have evolved a suite of sophisticated drought-resistance mechanisms, primarily including: (1) reduction in water transpiration through stomatal closure and cuticular wax accumulation; (2) enhancement of water acquisition capacity through root growth and architectural remodeling; (3) maintenance of cellular osmotic potential and turgor pressure through the synthesis and accumulation of osmolytes such as proline, soluble sugars, and betaine; (4) mitigation of oxidative damage through the activation of antioxidant systems, including enzymatic components (superoxide dismutase [SOD], peroxidase [POD], catalase [CAT]) and non-enzymatic components (ascorbic acid, glutathione) to scavenge reactive oxygen species (ROS); and (5) coordinated regulation of downstream responsive gene expression through hormone signaling pathways, particularly abscisic acid (ABA), jasmonic acid (JA), and salicylic acid (SA) [6,7,8]. In drought resistance research on sweet potato and other tuberous crops (e.g., potato, cassava), the application of omics technologies has similarly achieved remarkable progress. Specifically, RNA sequencing has emerged as a powerful tool for identifying drought-responsive genes and regulatory pathways [9]. For instance, transcriptomic analyses of leaves and roots from sweet potato cultivars with contrasting drought resistance have identified numerous differentially expressed genes (DEGs) under drought stress, which are extensively involved in the aforementioned biological processes [10]. Studies have revealed that many signal transduction-related genes, including protein kinases, protein phosphatases, and calcium signaling components, are rapidly activated during the early stages of drought response [11]. Concurrently, the expression of numerous functional protein genes, such as late embryogenesis abundant (LEA) proteins, aquaporins (AQPs), and key enzymes in various metabolic pathways including the flavonoid biosynthesis pathway, is significantly modulated in response to drought stress [12,13,14]. Recent investigations have further illuminated the roles of specific gene families in sweet potato drought resistance; for example, members of the *IbDUF668* gene family exhibit significant upregulation under drought treatment and may positively regulate drought tolerance in sweet potato [15]. Additionally, transcriptomic analyses of root development and stress responses in sweet potato have identified homeodomain family members, including *CYP450*, *KNOX*, and *Polygalacturonase* (*PG*) homologs, participating in drought stress responses [16,17,18]. While these studies have provided preliminary molecular insights into sweet potato drought resistance, many critical regulatory nodes and their intricate networks remain to be thoroughly investigated.

Transcription factors (TFs) function as pivotal regulatory proteins that activate or repress target gene transcription when plants encounter drought stress. Multiple TF families, including MYB, etc., have been established as core constituents in plant drought-resistance regulation [19,20]. Extensive research has demonstrated that MYB TFs play crucial roles in regulating plant secondary metabolism and responses to drought stress [21,22]. For example, in *Arabidopsis thaliana*, AtMYB11, AtMYB12, and AtMYB111 can regulate flavonol biosynthesis by directly activating early flavonoid genes such as CHS, CHI, F3H, and FLS [23,24,25]. Moreover, *AtMYB20* and *AtMYB60* influence drought resistance by modulating ABA signaling pathways and stomatal aperture, respectively [26,27]. In rice (*Oryza sativa*), MYB TFs can act as positive or negative regulators of drought resistance; for example, OsMYB1R1 negatively regulates drought resistance, whereas OsMYB2 positively regulate it [28,29]. Research on the MYB family in sweet potato has also advanced considerably. Notably, overexpression of *IbMYB330* in transgenic tobacco enhanced CAT and POD activities, thereby improving tolerance to drought treatment [30]. Additionally, overexpression of *IbMYB48* and *IbMYB116* could significantly enhance transgenic plant tolerance to drought stress [31,32]. Molecular mechanism analyses indicate that *IbMYB48* and *IbMYB116* may synergistically enhance stress tolerance by activating ABA biosynthesis pathways, jasmonic acid signaling pathways, and ROS scavenging systems. Recent studies have demonstrated that overexpression of *IbMYC2* can activate genes encoding ROS-scavenging enzymes and proline synthases, and enhance the expression of anthocyanin biosynthesis pathway genes by binding to the promoters of *IbCHI* and *IbDFR*, thereby conferring enhanced drought resistance [33]. These studies collectively establish MYB TFs as promising candidate genes for genetic improvement of drought resistance in sweet potato.

Although the functions of some *IbMYB* genes have been preliminarily validated, within the vast MYB family of sweet potato, whether additional undiscovered members play roles in drought-resistance regulatory networks, through which downstream pathways they exert their functions, and whether they interact with downstream structural genes remain unclear. Therefore, this study aims to identify the molecular mechanisms of sweet potato drought resistance through an integrated research strategy combining dynamic transcriptomic analysis and gene functional exploration. In the present study, an MYB-type TF previously identified in our preliminary screening [34] was subjected to systematic phylogenetic analysis to elucidate its taxonomic classification and infer its potential biological function. Subsequently, we performed RNAi- and overexpression-based functional validation of the screened MYB TF to elucidate its specific roles in sweet potato drought resistance and their underlying molecular mechanisms. This study aspires to provide novel insights into sweet potato drought-resistance molecular mechanisms, discover new genes with significant breeding value and provide useful genetic resources for future genetic improvement of drought resistance in sweet potato.

## 2. Results

### 2.1. Expression Profiling of IbMYB11

**Figure 1:**
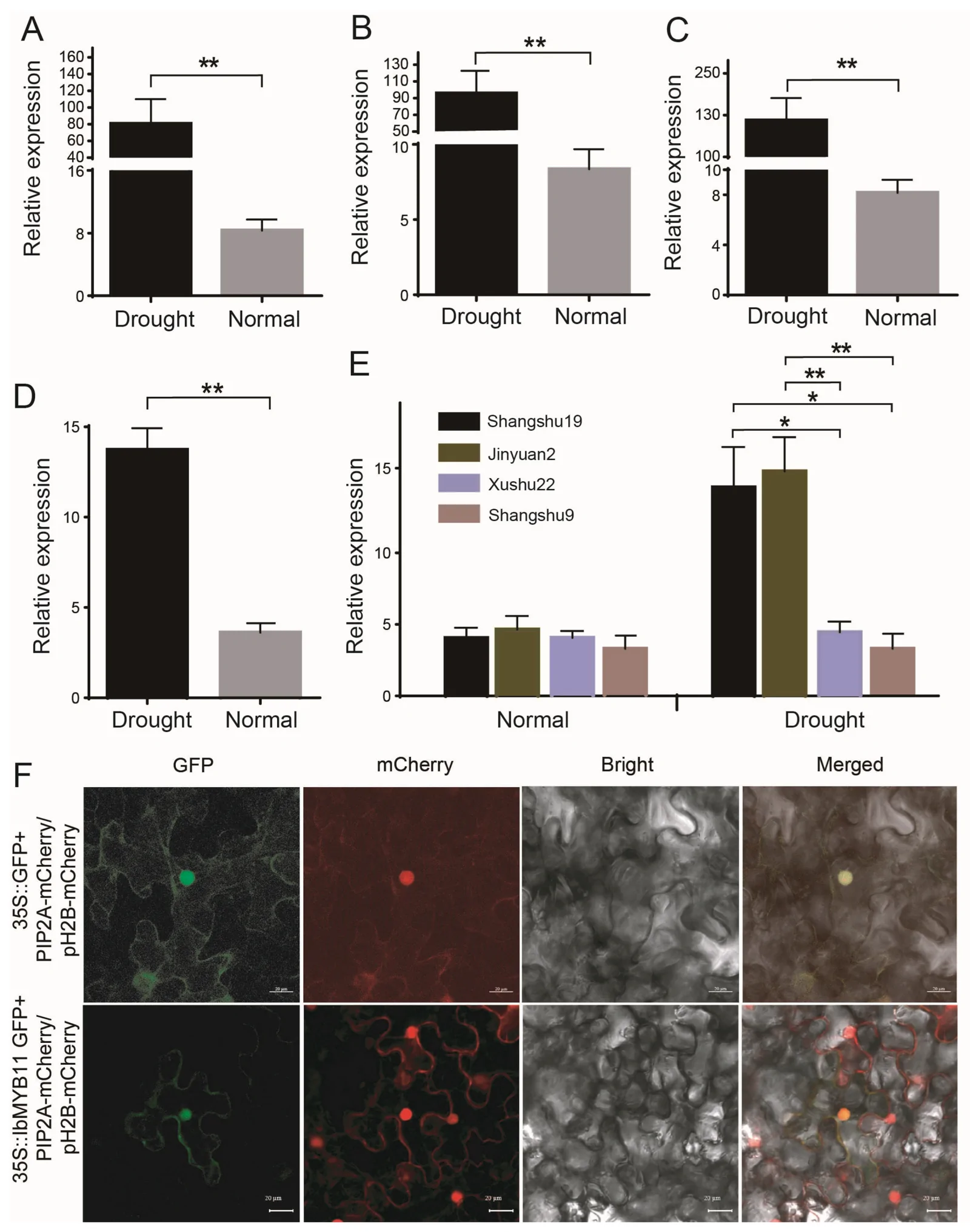
The response and expression pattern of *IbMYB11* to drought stress. Expression levels of *IbMYB11* in the leaves (**A**), stems (**B**), and roots (**C**) of Shangshu19 under natural drought treatment. (**D**) Expression of *IbMYB11* in the leaves of Shangshu19 under Normal and PEG (30%) treatment (Drought). (**E**) Expression of *IbMYB11* in the leaves of representative drought-resistant accessions (Shangshu19 and Jinyuan2) and representative drought-sensitive accessions (Xushu22 and Shangshu9) under Normal and PEG (30%) treatment (Drought). (**F**) Subcellular localization of IbMYB11 in tobacco leaves. Scale bars = 20 μm. For all RT-qPCR analyses, the control treatment was set to 1, and data are presented as mean ± SE of three biological replicates. Student’s *t*-test was used to determine significance: * *p* < 0.05; ** *p* < 0.01.

To determine the classification of the IbMYB, a total of 169 distinct MYB transcription factors were employed for phylogenetic reconstruction. Phylogenetic analysis clustered IbMYB from *Ipomoea batatas* with *Arabidopsis thaliana*’s *AtMYB11*, *AtMYB12*, *AtMYB111*, *AtMYB75*, *AtMYB114*, and *AtMYB123* into a single clade (Figure S1), which have been reported to regulate of flavonoid biosynthesis. Given that the IbMYB identified in this study represents a putative regulator of flavonoid biosynthesis like *AtMYB11*, we designated this gene as *IbMYB11*. *IbMYB11* was significantly upregulated in the roots, stems, and leaves of sweet potato under drought stress, with the highest fold change detected in the roots (Figure 1A–C). PEG-simulated drought treatment also strongly induced *IbMYB11* expression (Figure 1D). Upon drought treatment, *IbMYB11*’s expression was significantly or highly significantly higher in the drought-resistant cultivars Shangshu19 and Jinyuan2 than in the drought-sensitive cultivars Shangshu9 and Xushu22 (Figure 1E). Subcellular localization analysis revealed that IbMYB11 was localized to the nucleus (Figure 1F), consistent with its canonical function as a TF.

### 2.2. Generation and Functional Exploration of IbMYB11 Transgenic Sweet Potato Lines Under Drought Stress

**Figure 2:**
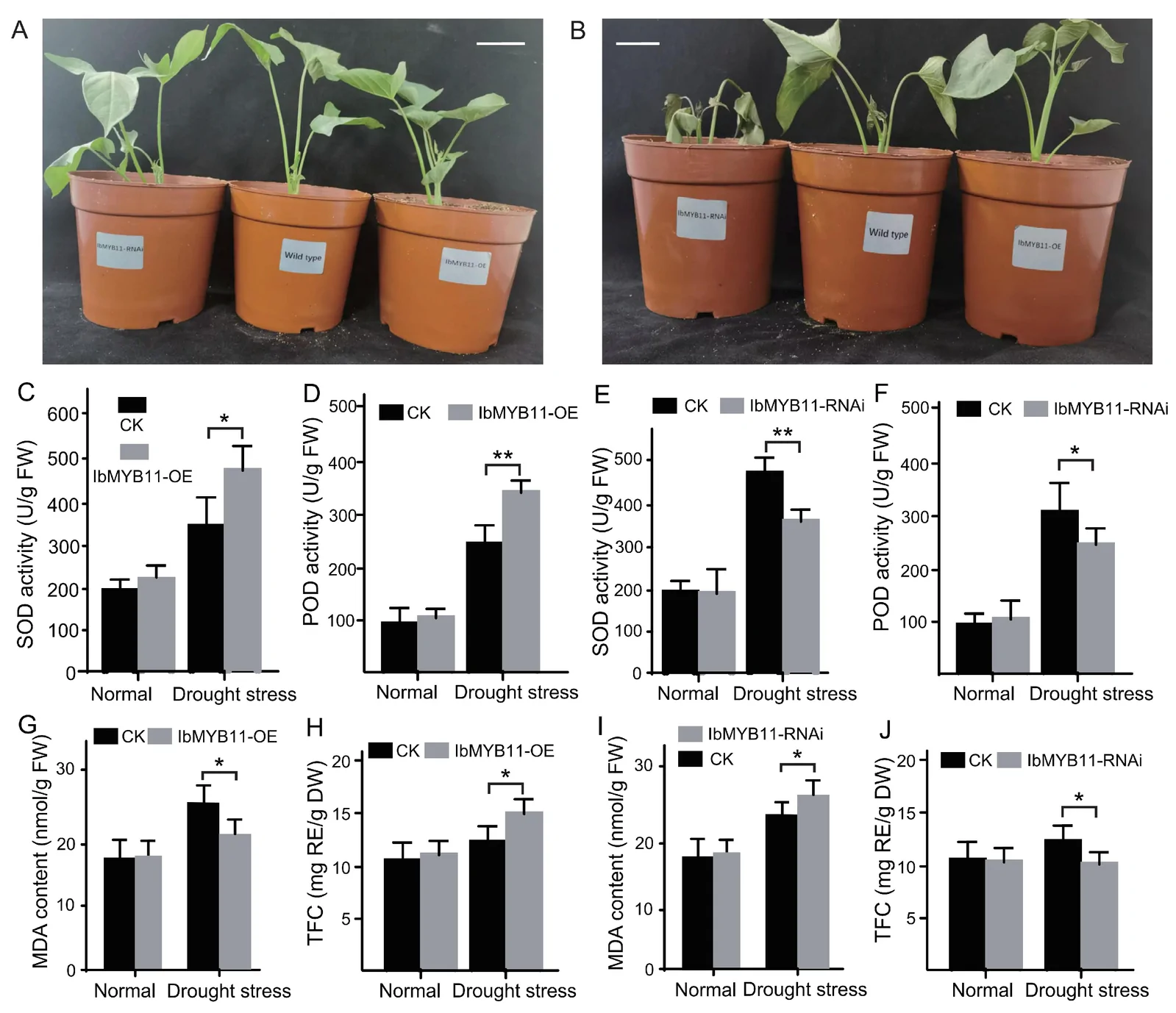
Phenotype and antioxidant enzyme activities in *IbMYB11*-transgenic plants under drought stress. (**A**) Phenotypes of *IbMYB11*-RNAi (left), wild-type (middle), and *IbMYB11*-OE (right) plants under normal conditions. Scale bars = 5 cm. (**B**) Phenotypes of *IbMYB11*-RNAi (left), wild-type (middle), and *IbMYB11*-OE (right) under PEG (30%) treatment for 72 h. Scale bars = 5 cm. (**C**) SOD activity in *IbMYB11*-OE and CK plants under drought stress. (**D**) POD activity in *IbMYB11*-OE and CK plants under drought stress. (**E**) SOD activity in *IbMYB11*-RNAi and CK plants under drought stress. (**F**) POD activity in *IbMYB11*-RNAi and CK plants under drought stress. (**G**) MDA content in *IbMYB11*-OE and CK plants under drought stress. (**H**) Total flavonoid content (TFC) in *IbMYB11*-OE and CK plants under drought stress. (**I**) MDA content in *IbMYB11*-RNAi and CK plants under drought stress. (**J**) TFC in *IbMYB11*-RNAi and CK plants under drought stress. For all physiological indicators, the data are presented as mean ± SE of three biological replicates. Student’s *t*-test was used to determine significance: * *p* < 0.05; ** *p* < 0.01.

Under drought stress, *IbMYB11* transcript levels were elevated by more than 5-fold in OE plants compared to wild-type controls (Figure S3A), whereas expression was reduced by approximately 50% in RNAi lines (Figure S3B). To further investigate the functional role of *IbMYB11*, wild-type, *IbMYB11*-OE, and *IbMYB11*-RNAi plants were subjected to 30% (*w*/*v*) PEG treatment. After 72 h, marked phenotypic differences were apparent between treated and untreated control plants. The *IbMYB11*-RNAi lines displayed severe whole-plant wilting, whereas WT plants also exhibited wilting but to a markedly lesser degree. By contrast, *IbMYB11*-OE plants showed only slight leaf wilting (Figure 2A,B). Subsequently, the activities of superoxide dismutase (SOD), peroxidase (POD), total flavonoid content (TFC) and MDA content were determined in transgenic and control plants subjected to three days of drought treatment. Both SOD, POD activities and TFC were significantly enhanced in *IbMYB11*-OE plants compared to controls (Figure 2C,D,H). Conversely, the two antioxidant enzyme activities and TFC were significantly reduced in *IbMYB11*-RNAi plants (Figure 2E,F,J). The MDA contents were significantly lower in OE the lines and higher in the RNAi lines (*p* < 0.05) (Figure 2G,I). Collectively, these results suggest that *IbMYB11* might enhance drought tolerance by upregulating antioxidant enzyme activities and downregulating ROS accumulation, in which flavonoids might be involved.

### 2.3. Transcriptome Analysis of IbMYB11-RNAi and IbMYB11-OE Plants

To further elucidate the genome-wide regulatory impact of *IbMYB11* expression, we conducted transcriptome sequencing of *IbMYB11*-OE, *IbMYB11*-RNAi, and control plants under simulated drought stress. Quality control metrics, including Q30 scores, sequencing depth, mapping rates, and overall transcript abundance, confirmed high sequencing quality (Table S2; Figures S4 and S5), enabling reliable downstream differential expression analysis.

**Figure 3:**
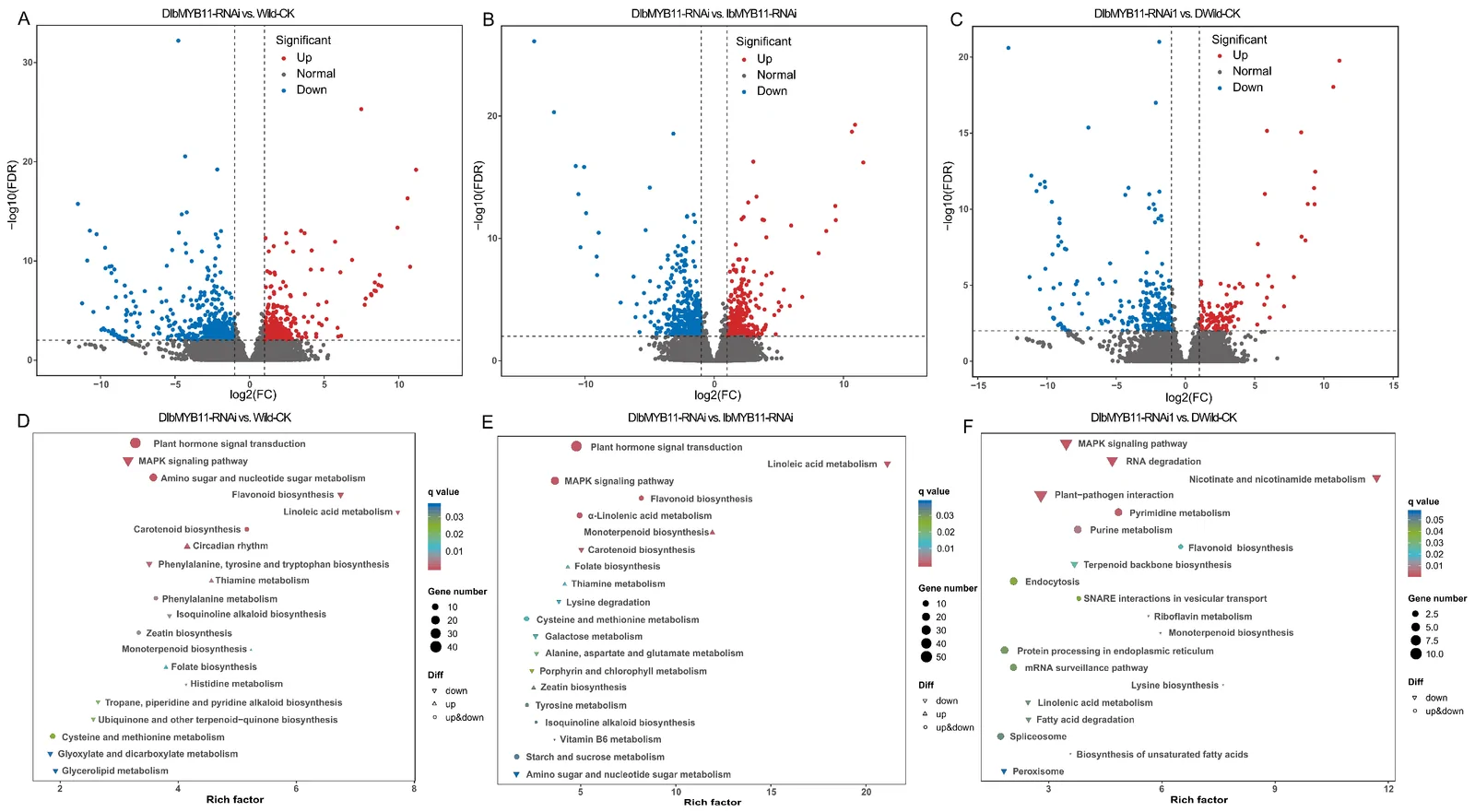
Differential expression ((**A**): comparison group D*IbMYB11*-RNAi vs. Wild-CK; (**B**): comparison group D*IbMYB11*-RNAi vs. *IbMYB11*-RNAi; (**C**): comparison group D*IbMYB11*-RNAi vs. DWild-CK) and enrichment analysis ((**D**): comparison group D*IbMYB11*-RNAi vs. Wild-CK; (**E**): comparison group D*IbMYB11*-RNAi vs. *IbMYB11*-RNAi; (**F**): comparison group D*IbMYB11*-RNAi vs. DWild-CK) of the *IbMYB11*-RNAi plants.

Differential expression analysis was performed by comparing PEG-simulated drought-stressed D*IbMYB11*-RNAi samples (6 h) with Wild-CK (0 h), DWild-CK (6 h), and *IbMYB11*-RNAi (0 h), yielding 816, 718, and 350 differentially expressed genes (DEGs), respectively (Figure S6A). KEGG pathway enrichment analysis revealed that in the D*IbMYB11*-RNAi vs. Wild-CK comparison group, the top five enriched pathways included “Plant hormone signal transduction”, “MAPK signaling pathway”, “Amino sugar and nucleotide sugar metabolism”, “Flavonoid biosynthesis” and “Linoleic acid metabolism”. In the D*IbMYB11*-RNAi vs. *IbMYB11*-RNAi comparison, the top five enriched pathways comprised “Plant hormone signal transduction”, “Linoleic acid metabolism”, “MAPK signaling pathway”, “Flavonoid biosynthesis” and “α-Linoleic acid metabolism”. By contrast, the D*IbMYB11*-RNAi vs. DWild-CK comparison showed enrichment of “MAPK signaling pathway”, “RNA degradation”, “Nicotinate and nicotinamide metabolism”, “Plant–pathogen interaction” and “Pyrimidine metabolism” among the top five pathways. Notably, the flavonoid biosynthesis pathway was consistently represented within the top five enriched pathways in both the D*IbMYB11*-RNAi vs. Wild-CK and D*IbMYB11*-RNAi vs. *IbMYB11*-RNAi comparisons. Although this pathway was not ranked among the top five in the D*IbMYB11*-RNAi vs. DWild-CK comparison, it remained enriched within the top ten pathways (Figure 3).

**Figure 4:**
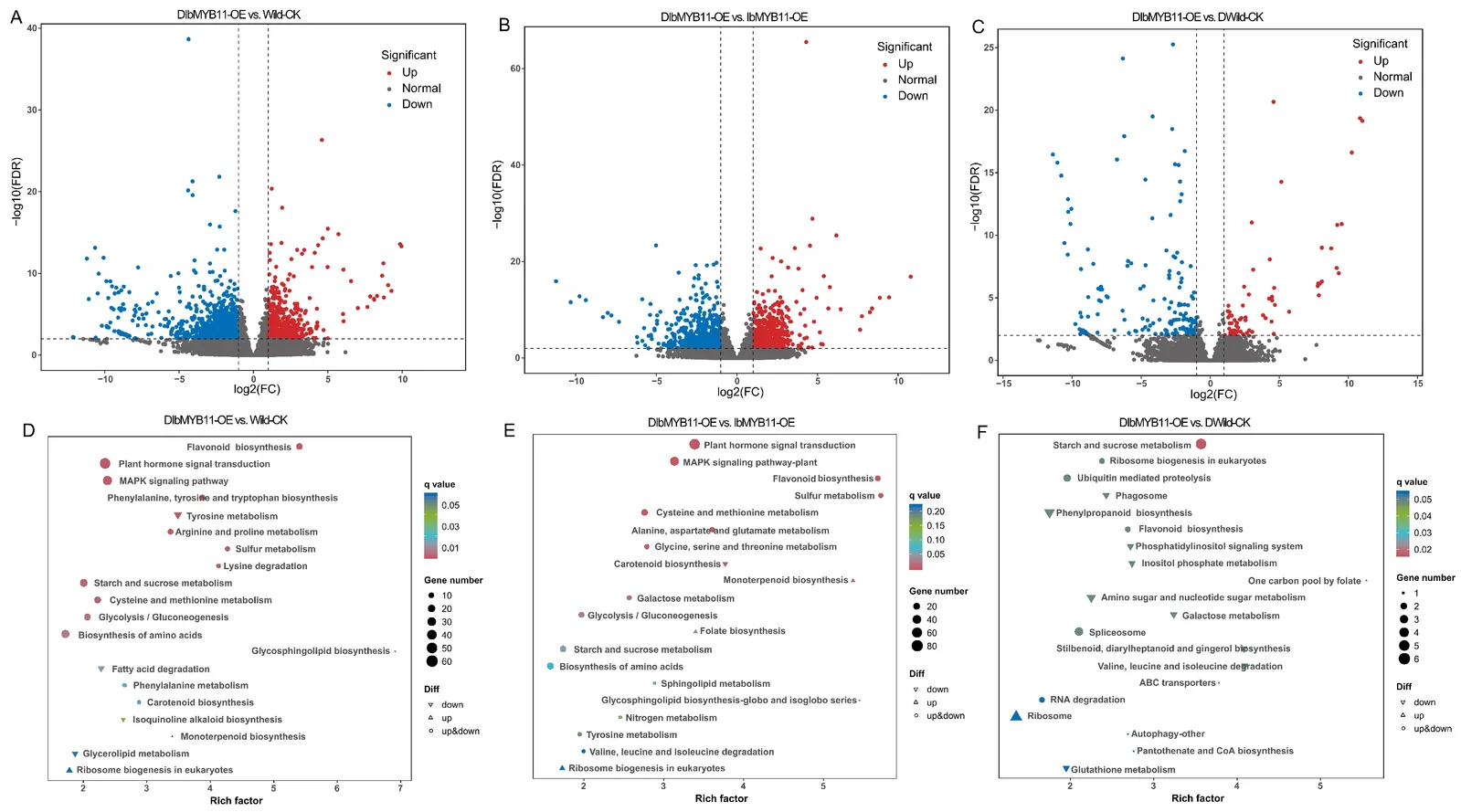
Differential expression ((**A**): comparison group D*IbMYB11*-OE vs. Wild-CK; (**B**): comparison group D*IbMYB11*-OE vs. *IbMYB11*-OE; (**C**): comparison group D*IbMYB11*-OE vs. DWild-CK) and enrichment analysis ((**D**): comparison group D*IbMYB11*-OE vs. Wild-CK; (**E**): comparison group D*IbMYB11*-OE vs. *IbMYB11*-OE; (**F**): comparison group D*IbMYB11*-OE vs. DWild-CK) of the *IbMYB11*-OE plants.

In parallel, transcriptome analysis was conducted for *IbMYB11*-OE samples. Comparisons of D*IbMYB11*-OE vs. Wild-CK, D*IbMYB11*-OE vs. *IbMYB11*-OE, and D*IbMYB11*-OE vs. DWild-CK identified 1593, 1668, and 230 DEGs, respectively (Figure S6B). KEGG enrichment analysis of DEGs from the D*IbMYB11*-OE vs. Wild-CK and D*IbMYB11*-OE vs. *IbMYB11*-OE comparisons revealed that, beyond “Plant hormone signal transduction” and “MAPK signaling pathway”, the flavonoid biosynthesis pathway was also commonly enriched. Moreover, the flavonoid biosynthesis pathway was enriched among DEGs in the D*IbMYB11*-OE vs. DWild-CK comparison (Figure 4). Collectively, these findings suggest that *IbMYB11* may participate in drought stress responses in sweet potato by modulating flavonoid biosynthesis.

### 2.4. Metabolomic Analysis of IbMYB11-RNAi and IbMYB11-OE Plants

**Figure 5:**
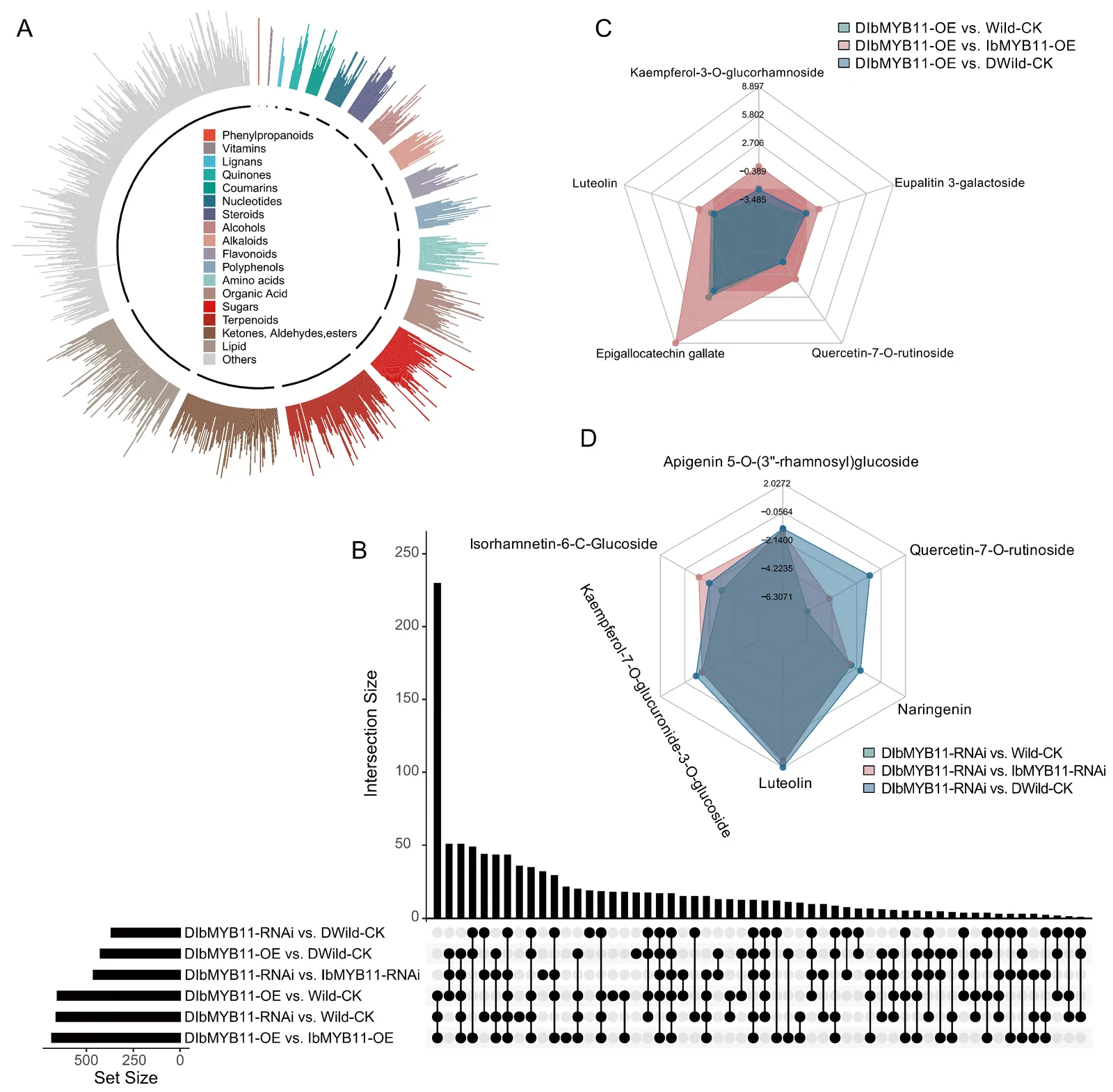
Differentially accumulated metabolites and KEGG enrichment analysis of *IbMYB11*-OE and *IbMYB11*-RNAi plants. (**A**) Classification of the metabolites identified by metabolomic profiling. (**B**) UpSet plot of differentially expressed metabolites (DEMs) across different comparison groups. (**C**) Radar chart of differentially expressed flavonoid metabolites in *IbMYB11*-OE plants. The numbers on the radar chart represent relative content values of flavonoid metabolites. (**D**) Radar chart of differentially expressed flavonoid metabolites in *IbMYB11*-RNAi plants.

To verify whether flavonoid metabolite contents differed in *IbMYB11*-RNAi and *IbMYB11*-OE samples, metabolomic profiling was performed synchronously using the same plant materials employed for the above transcriptome sequencing. Principal component analysis (PCA) of metabolite quantification data demonstrated acceptable consistency among biological replicates (Figure S7). A total of 17 primary metabolite categories were detected across all samples, including flavonoids and phenylpropanoids, encompassing 1039 annotated metabolites (Figure 5A). Subsequently, differential metabolite analysis (DEMs) was conducted across six comparison groups consistent with the transcriptomic dataset (Figure S8), followed by upset plot visualization (Figure 5B).

Among the DEMs shared across the three comparison groups—D*IbMYB11*-OE vs. Wild-CK, D*IbMYB11*-OE vs. *IbMYB11*-OE, and D*IbMYB11*-OE vs. DWild-CK—five flavonoid metabolites were identified: kaempferol-3-O-glucorhamnoside, eupalitin 3-galactoside, phlorizin, epigallocatechin gallate, and 2″-O-rhamnosylicariside II (Figure 5C). Drought stress promoted the accumulation of epigallocatechin gallate and 2″-O-rhamnosylicariside II in D*IbMYB11*-OE plants. Similarly, among the DEMs shared across the three comparison groups—D*IbMYB11*-RNAi vs. Wild-CK, D*IbMYB11*-RNAi vs. *IbMYB11*-RNAi, and D*IbMYB11*-RNAi vs. DWild-CK—six flavonoid metabolites were identified: naringenin, luteolin, isorhamnetin-6-C-glucoside, quercetin-7-O-rutinoside, kaempferol-7-O-glucuronide-3-O-glucoside, and apigenin 5-O-(3″-rhamnosyl) glucoside (Figure 5D). Notably, the accumulation of naringenin, kaempferol-7-O-glucuronide-3-O-glucoside, and apigenin 5-O-(3″-rhamnosyl) glucoside was suppressed in D*IbMYB11*-RNAi plants under drought stress. Among the shared metabolites, luteolin and quercetin-7-O-rutinoside showed increased accumulation in the *IbMYB11*-OE plants, whereas their levels decreased in the *IbMYB11*-RNAi plants. Collectively, these results indicate that genetic manipulation of *IbMYB11* modulates flavonoid-related metabolite accumulation in sweet potato under drought stress.

### 2.5. Weighted Gene Co-Expression Network Analysis (WGCNA) Based on Co-Expression Module and Flavonoid Metabolites

**Figure 6:**
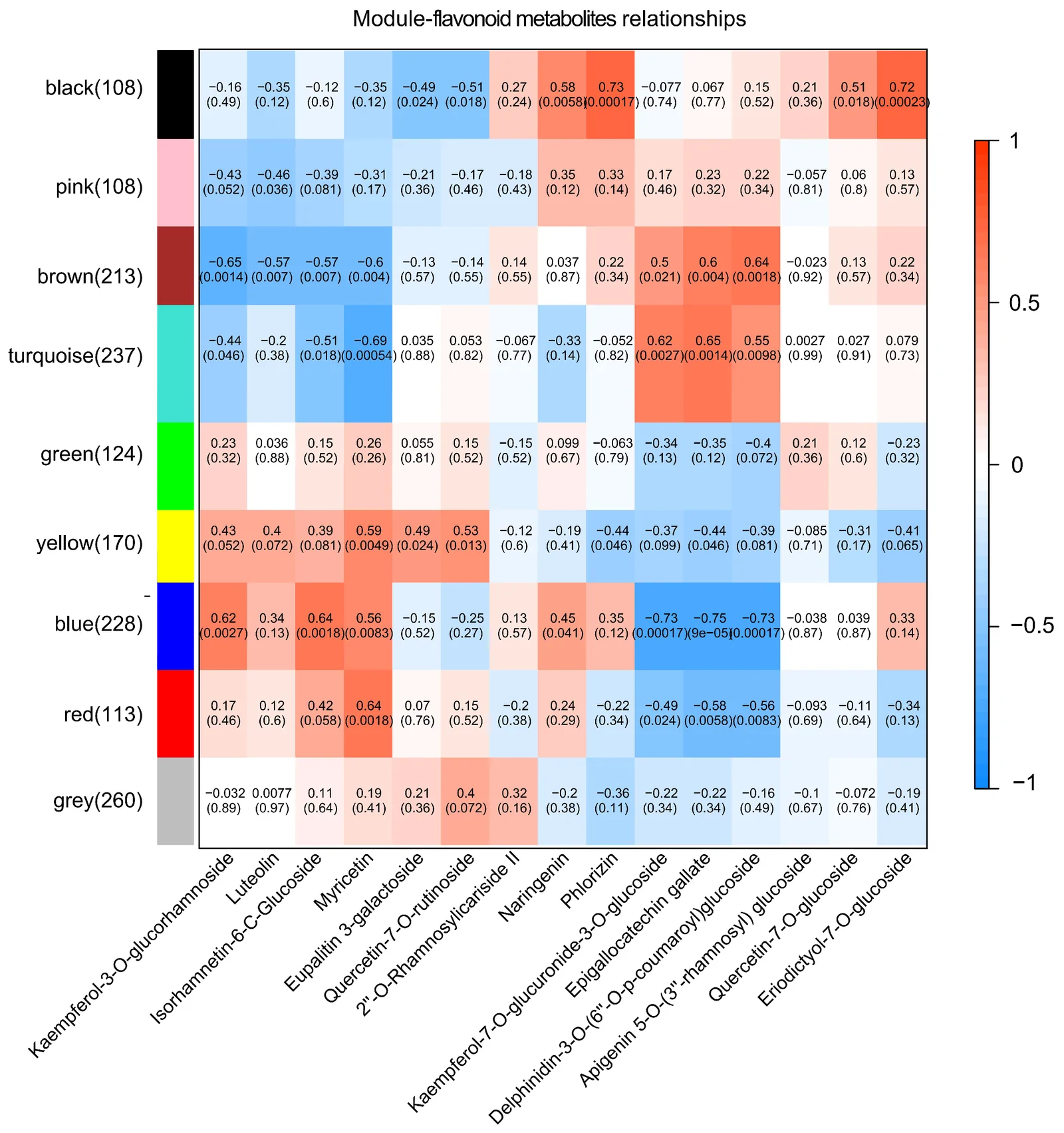
The correlation between co-expression modules and flavonoid metabolites using WGCNA. The different colors on the left represent different coexpression modules. In each correlation block, the number at the top indicates the correlation values, and the corresponding *p* values are indicated in parentheses below.

To further dissect the potential regulatory relationships between transcription factors (TFs) and flavonoid accumulation, we identified a total of 1735 TFs and 15 flavonoid metabolites, which were subsequently subjected to WGCNA. The 1735 TFs were assigned to nine co-expression modules, from which 24 hub TFs were identified, with NAC, WRKY, and bHLH representing the most abundant families (Table S3). Notably, *IbMYB11* was a hub gene in the black module. In total, 15 module–metabolite pairs met the significance threshold (|correlation coefficient| ≥ 0.6 and *p* < 0.05). The strongest positive correlation was observed between the black module and phlorizin (r = 0.73, *p* = 1.7 × 10^−4^), followed closely by the correlation between the black module and eriodictyol-7-O-glucoside (r = 0.72, *p* = 2.3 × 10^−4^). The strongest negative correlation (r = −0.75, *p* = 9 × 10^−5^) was detected between the blue module and epigallocatechin gallate. Notably, the blue module was significantly correlated with as many as five distinct flavonoid metabolites (kaempferol-3-O-glucorhamnoside, isorhamnetin-6-C-glucoside, kaempferol-7-O-glucuronide-3-O-glucoside, epigallocatechin gallate, and delphinidin-3-O-(6′′-O-p-coumaroyl) glucoside) (|r| ≥ 0.6, *p* ≤ 0.05) (Figure 6). Collectively, these results suggest that, in addition to MYB-type factors, multiple TF families act in concert to regulate flavonoid accumulation under drought stress.

## 3. Discussion

### 3.1. Transcription Factor Network-Mediated Drought Resistance in Sweet Potato

Based on previously screened results [34], this study unequivocally established that a novel MYB-type TF *IbMYB11* plays a critical positive regulatory role in sweet potato drought resistance. Our RNAi and overexpression experimental results consistently demonstrated that the expression level of *IbMYB11* is positively correlated with drought tolerance in sweet potato. Transgenic lines overexpressing this gene exhibited higher protective enzyme activities under drought stress, whereas RNAi lines showed opposite phenotypes. This discovery not only adds a new key member to the molecular mechanisms of sweet potato drought resistance but also highlights the universal importance of the MYB family in plant stress responses. Previous reports indicated that *IbMYB2* primarily enhances drought resistance by regulating anthocyanin synthesis to strengthen antioxidant capacity [33], while *IbMYB48* and *IbMYB116* are considered more upstream regulatory factors potentially functioning through the integration of multiple hormone signaling pathways including ABA and JA [31,32]. *IbMYB11*, based on its downstream regulatory pathways, appears to focus more on regulating flavonoid metabolism to relieve oxidative damage induced by drought stress (Figure 2). These results suggest the existence of complex regulatory mechanisms comprising multiple MYB TFs in sweet potato. During the process of responding to drought, different MYB family members may exhibit functional division and cooperation: some members (such as *IbMYB48* and *IbMYB116*) may be responsible for perceiving and transmitting upstream signals, while others (such as *IbMYB2* and our discovered *IbMYB11*) are responsible for executing specific downstream responses. This functional specialization and coordination enable plants to respond precisely and efficiently to drought stress of varying intensity and duration.

However, plant drought resistance is an extremely complex trait. In the transcriptomic data of this study, we also identified other types of transcription factors, such as differentially expressed members of the NAC, bZIP, WRKY, and AP2/ERF families (Figure 6). Based on enrichment analysis and WGCNA performed on the transcriptomic data from this study, we discovered that the expression patterns of certain TF families exhibit highly significant positive or negative correlations with multiple key structural genes in the flavonoid biosynthesis pathway (Table S4). Therefore, future research will encompass systematic functional characterization of these candidate genes and exploration of potential crosstalk between them and the *IbMYB* regulatory pathway. We speculate that *IbMYB11* does not function in isolation but serves as an important node within complex regulatory networks, acting synergistically with other transcription factors to regulate sweet potato drought resistance. This synergistic interaction may be achieved through multiple mechanisms: first, forming transcriptional cascades, wherein one TF regulates the expression of another, establishing upstream–downstream relationships; second, forming transcriptional complexes through protein–protein interactions that jointly bind to promoters of downstream target genes, achieving fine-tuned regulation of gene expression. For instance, MYB TFs often require interaction with bHLH and WD40 proteins to form MBW complexes for regulating downstream genes [35]. Consequently, an important future research direction involves dissecting the position of *IbMYB11* within the entire transcriptional regulatory network, identifying its upstream and downstream regulatory factors and regulated target genes, with the ultimate goal of constructing a comprehensive molecular regulatory network for sweet potato drought resistance spanning signal perception, signal transduction, transcriptional regulation, and physiological responses, thereby providing solid theoretical foundations and a rich toolbox of genes for molecular design breeding in sweet potato.

### 3.2. IbMYB11 May Regulate Drought Resistance in Sweet Potato by Mediating the Flavonoid Biosynthesis Pathway

Transcriptomic analysis revealed that a total of 23 structural genes associated with the flavonoid biosynthesis pathway were differentially expressed, encompassing members of the ANS, UGT, CHIL, PAL, CHS, F3H, and C4H families. Under drought stress, *Ibat.Tzn_v3.12EG026830.1* (CHIL), *Ibat.Tzn_v3.06BG026580.1* (UGT), *Ibat.Tzn_v3.01AG030070.1* (CHS), *Ibat.Tzn_v3.04CG000440.2* (UGT), *Ibat.Tzn_v3.09AG015310.1* (PAL), and *Ibat.Tzn_v3.04BG000380.1* (UGT) exhibited downregulated expression in IbMYB11-RNAi plants, whereas *Ibat.Tzn_v3.13AG005650.1* (ANS), *Ibat.Tzn_v3.04BG000380.2* (UGT), *Ibat.Tzn_v3.04AG000360.1* (UGT), *Ibat.Tzn_v3.04CG000440.1* (UGT), *Ibat.Tzn_v3.09EG015880.1* (PAL), *Ibat.Tzn_v3.04FG000420.1* (UGT), *Ibat.Tzn_v3.04CG000440.2* (UGT), and *Ibat.Tzn_v3.04BG000380.1* (UGT) showed upregulated expression in IbMYB11-OE plants (Table S4). These findings suggest that IbMYB11 may modulate flavonoid accumulation by regulating these flavonoid pathway structural genes in sweet potato under drought stress. Previous studies have demonstrated that AtMYB11, AtMYB12, and AtMYB111 collaboratively activate the expression of early flavonoid pathway structural genes, including CHS, CHI, F3H, and FLS1 [36], indicating that IbMYB11 may play a role in flavonoid biosynthesis. Flavonoids represent powerful non-enzymatic antioxidants in plants. Under drought conditions, plants experience bursts of reactive oxygen species (ROS) production, leading to membrane lipid peroxidation, protein denaturation, and DNA damage. Flavonoids can effectively scavenge excess ROS, maintain intracellular redox homeostasis, and thereby protect cellular structural and functional integrity—an important mechanism for plant stress adaptation [37]. In multiple plant species, MYB-type TFs serve as core regulatory elements controlling flavonoid biosynthesis pathways. They can directly bind to MYB-binding sites within the promoter regions of genes such as *CHS* to activate their transcription [38,39]. In sweet potato, *IbMYB1* has been established as the key factor regulating anthocyanin accumulation in purple-fleshed storage roots [40,41]. Moreover, Sweet potato IbMYB340–IbbHLH2–IbERF71 complex targets the IbANS1 promoter and regulates anthocyanin biosynthesis in purple-fleshed sweet potato [42]. Our study links the *IbMYB11* gene to flavonoid synthesis in response to drought stress, highlighting the extensive involvement of MYB TFs in the regulation of metabolite biosynthesis. The higher antioxidant enzyme activities (SOD and POD) in *IbMYB11*-overexpressing lines and the lower activities in *IbMYB11*-RNAi plants align with the enhanced antioxidant capacity expected to result from flavonoid accumulation. This provides functional evidence supporting our hypothesis, delineating a complete molecular mechanism from *IbMYB11* upregulation, through flavonoid pathway activation and enhanced antioxidant capacity, to improved plant drought resistance.

### 3.3. Potential Interacting Protein Network of IbMYB11 and Future Research Perspectives

To elevate our understanding of *IbMYB11* function from a “linear pathway” to a “regulatory network” level, the key is to identify its potential interacting proteins. As previously discussed, MYB TFs typically require the formation of MBW (MYB-bHLH-WD40) ternary protein complexes to regulate flavonoid synthesis pathways. *IbMYB11* likely requires interaction with specific IbHLH and IbWD40 proteins in sweet potato to efficiently exert its transcriptional activation function, particularly given that we observe positive correlations in expression between *IbMYB11* and certain *IbHLH* and *IbWD40* genes (correlation coefficient = 0.81, *p* < 0.01). It should be noted that whether IbMYB11 directly binds to the promoters of flavonoid biosynthetic genes remains to be determined. Moving forward, we plan to investigate these potentially positively correlated bHLH and *WD40*-encoding genes as subsequent targets for interaction studies, utilizing yeast two-hybrid (Y2H) systems or bimolecular fluorescence complementation (BiFC) technologies. Second, to demonstrate whether *IbMYB11* directly regulates flavonoid pathway genes, we will employ yeast one-hybrid (Y1H), electrophoretic mobility shift assay (EMSA), and dual-luciferase reporter assays to verify whether the *IbMYB11* protein can specifically bind to promoter regions of downstream target genes such as *CHS* and *UGT* and activate their transcriptional activity. Finally, chromatin immunoprecipitation sequencing (ChIP-seq) or DNA affinity purification sequencing (DAP-seq) could be employed to analyze the genome-wide binding sites of the *IbMYB11* protein in sweet potato, ultimately confirming the functional elements of *IbMYB11* in sweet potato drought resistance and elucidating the interrelationships between *IbMYB11* and its target genes.

## 4. Materials and Methods

### 4.1. Construction of the MYB TF Evolutionary Tree

Protein sequences of all Arabidopsis MYB TFs were retrieved from the Plant Transcription Factor Database v5.0 (https://planttfdb.gao-lab.org) and combined with the IbMYB protein sequences. Multiple sequence alignment was performed using MUSCLE5 [43], and the resulting alignment was trimmed with trimAL [44] to remove ambiguously aligned regions. Maximum likelihood phylogenetic inference was conducted using IQ-TREE2 with 1000 bootstrap replicates [45]. The resulting tree was visualized and annotated using the iTOL online tool (https://itol.embl.de/).

### 4.2. Cloning and Genetic Transformation of IbMYB11

The sweetpotato cultivar Shangshu 19 was used for gene cloning and expression analysis. Plants of Xushu 19 were grown in a controlled greenhouse at 28/22 °C (day/night), 16 h/8 h photoperiod, 60–70% relative humidity, and a light intensity of 400 μmol m^−2^*s^−1^. Plants were grown in plastic pots (20 cm diameter × 25 cm height) filled with a 3:1:1 mixture of garden soil, peat moss, and perlite (*v*/*v*/*v*). Total RNA was extracted from young leaves using the plant total RNA extraction kit (Tiangen, Wuhan, China), followed by quality and quantity checks using 1% agarose gel electrophoresis and Nano-Drop ND-1000 spectrophotometer (Thermo Fisher Scientific, Wilmington, MA, USA). First-strand cDNA was synthesized from 1.0 μg total RNA using the HiFiScript gDNA Removal RT MasterMix kit (CWBIO, Taizhou, China) according to the manufacturer’s instructions. The reaction was performed in a 10 μL volume on ice. The amplified products were mixed with 6× loading buffer and separated by agarose gel electrophoresis. Target bands were excised under a gel documentation system and purified using the Gel Extraction Kit (CWBIO, Taizhou, China). The purified DNA was eluted with 50 μL Buffer EB and collected by centrifugation at 3000 rpm for 2 min, and stored at −20 °C.

The overexpression vector pCambia1300-35S-*IbMYB11* [46] was constructed by inserting the full-length CDS of *IbMYB11* downstream of the CaMV35S promoter. For RNA interference (RNAi), a fragment located within the non-conserved domain of *IbMYB11* was amplified and cloned into the pFGC5941 vector according to Hu et al. [33]. Primers were designed using Primer3 (https://www.primer3plus.com/index.html, accessed on 1 April 2026). The recombinant vectors were introduced into *Agrobacterium tumefaciens* strain EHA105 [47]. Positive clones were selected on LB agar supplemented with kanamycin and rifampicin. A single colony was inoculated into liquid LB medium containing the same antibiotics and incubated at 28 °C with shaking at 200 rpm for 14 h to reach the logarithmic growth phase. The bacterial suspension was then diluted with an equal volume of sterile distilled water prior to inoculation. Two-month-old field-grown stem cuttings of Shangshu 19 were collected as explants. Stem segments (~30 cm, six nodes) were excised and maintained in distilled water. An *A. tumefaciens*-mediated in planta transformation approach was employed for genetic transformation [48]. Briefly, the nodal regions, enriched with highly active meristematic cells, were targeted for transformation. Approximately 20 micro-wounds were uniformly created at each nodal region using a syringe needle to facilitate *Agrobacterium* infiltration. The wounded segments were immersed in the diluted *Agrobacterium* suspension, ensuring complete contact of the wounded surfaces with the bacterial solution, and incubated in the dark for 12 h. After inoculation, the segments were blotted with sterile filter paper to remove excess bacteria, washed five times with sterile distilled water, and immersed in 150 mg/L cefotaxime solution for 30 min to suppress residual Agrobacterium. After rinsing, the segments were directly transplanted into the field for recovery and tuber development. Genomic DNA was extracted from putative transgenic tubers, and the transgenic sweet potato lines overexpressing (OE) or silencing (RNAi) *IbMYB11* were verified by PCR (Table S1; Figure S2), with ddH_2_O and wild-type Shangshu 19 genomic DNA as negative controls. Positive tubers were planted in soil, and the regenerated shoots were subjected to secondary PCR screening. Confirmed positive plants were transplanted to the field, and stable transgenic lines were established through successive propagation and verification. The same primer pairs were used for both tuber and shoot identification.

### 4.3. RT-qPCR Analysis of IbMYB11

A 30% (*w*/*v*) PEG (polyethylene glycol) 6000 solution was prepared by dissolving 300 g of PEG 6000 in distilled water with continuous stirring and gentle heating (40–50 °C) until completely dissolved, and the final volume was adjusted to 1 L with distilled water. Transgenic sweetpotato lines overexpressing *IbMYB11* (OE), RNAi-silenced lines, and wild-type (WT) Shangshu 19, Shangshu9, Jinyuan2 and Xushu22 were grown under well-watered conditions until 4–5 leaf stage, and subjected to drought stress under 30% PEG 6000 for 6 h. Briefly, all plants were grown in pots containing an equal weight of the same substrate and were sufficient irrigated with an equal volume of water before the treatment, so that every pot started from a uniform saturated water status. The 30% (*w*/*v*) PEG 6000 solution was then applied to all pots (~500 mL/pot) simultaneously at 7 days after the last irrigation, with an equal and sufficient volume applied evenly onto the substrate surface in several aliquots to ensure uniform infiltration. Total RNA extraction and cDNA synthesis were performed as described above. RT-qPCR was conducted using SYBR Green Master Mix on a real-time PCR system in a 20 μL reaction volume containing 10 μL 2× master mix, 0.4 μM each of gene-specific forward and reverse primers, and 2 μL diluted cDNA template. The amplification program consisted of an initial denaturation at 95 °C for 30 s, followed by 40 cycles of 95 °C for 10 s and 60 °C for 30 s. Melting curve analysis was performed to confirm single-product amplification. The IbActin gene served as the internal reference, and relative expression levels were calculated using the 2^−ΔΔCt^ method [49]. All experiments were performed with three biological replicates and three technical replicates.

### 4.4. Subcellular Localization of IbMYB11

For subcellular localization analysis, the full-length coding sequence (CDS) of *IbMYB11* excluding the stop codon was amplified from sweet potato Xuzi 8 cDNA using Phusion High-Fidelity DNA Polymerase and gene-specific primers using primer3 (https://www.primer3plus.com/index.html) (Table S1). The purified amplicon was cloned into the multiple cloning site of the pYBA1132-GFP vector [50], generating the 35S::*IbMYB11*-GFP in-frame fusion construct driven by the cauliflower mosaic virus (CaMV) 35S promoter. The empty pYBA1132-GFP vector served as a negative control. All constructs were sequence-verified and subsequently introduced into *Agrobacterium tumefaciens* strain GV3101 [51] by the heat-shock (freeze–thaw) method. Positive transformants were selected on solid LB agar supplemented with 50 μg mL^−1^ kanamycin and 50 μg mL^−1^ rifampicin, and confirmed by colony PCR. For infiltration, individual positive clones were cultured in 50 mL liquid LB medium containing the same antibiotics at 28 °C with constant shaking (200 rpm) until the OD_600_ reached approximately 0.6. The bacterial cells were harvested by centrifugation at 4 °C and 12,000 rpm for 10 min, washed once, and resuspended in an infiltration buffer containing 10 mM MgCl_2_, 10 mM MES (pH 5.6), and 100 μM acetosyringone (AS) to a final OD_600_ of 0.6. *A. tumefaciens* strains harboring PIP2A-mCherry (plasma membrane marker) [52] and H2B-mCherry (nuclear marker) [53] were activated using the identical procedure. After incubation at room temperature for 4 h, the bacterial suspensions carrying the target constructs and the respective organelle markers were mixed at a 1:1 ratio and co-infiltrated into the abaxial side of fully expanded leaves of 4–5-week-old *Nicotiana benthamiana* plants using a needleless syringe. The infiltrated plants were kept in darkness for 24 h, followed by cultivation under normal growth conditions (16 h light/8 h dark at 22 °C) for an additional 24 h. Leaf discs from the infiltration zones were excised and immediately observed under a laser scanning confocal microscope (LSM 880, Carl Zeiss Jena, Jena, Germany) with excitation/emission wavelengths set at 488 nm/505–530 nm for GFP and 561 nm/580–620 nm for mCherry.

### 4.5. Determination of Antioxidant Enzyme Activity, Malonaldehyde (MDA) and Total Flavonoid Content (TFC) in Genetically Transformed Plants Under Drought Stress

*IbMYB11*-OE, *IbMYB11*-RNAi, and wild-type (Shangshu 19) were grown under well-watered conditions until 4–5 leaf stage, and subjected to drought stress under 30% PEG (polyethylene glycol) for 72 h. Leaf samples were collected, flash-frozen in liquid nitrogen, and stored at −80 °C for subsequent biochemical and molecular analyses. Frozen leaf tissues (0.1 g) were ground to a fine powder in liquid nitrogen and homogenized in 1 mL ice-cold extraction buffer [50 mM sodium phosphate (pH 7.8), 0.1 mM EDTA, 1% (*w*/*v*) polyvinylpyrrolidone (PVP)]. The homogenate was centrifuged at 10,000× *g* for 15 min at 4 °C, and the supernatant was collected for enzyme determination. Superoxide dismutase (SOD), peroxidase (POD) activities and MDA content were measured according to previously published methods [31,54]. The TFC was determined according to the method described by Jing et al. [55].

### 4.6. Flavonoid Pathway Structure Gene Annotation

Putative structural genes involved in the flavonoid biosynthetic pathway were identified from the sweetpotato reference genome (SweetGAINS I. batatasTanzania Phased v3, https://sweetpotato.uga.edu/) by a combined homology search and conserved domain validation strategy. Protein sequences of known flavonoid biosynthetic enzymes, including chalcone synthase (CHS), chalcone isomerase (CHI), flavanone 3-hydroxylase (F3H), flavonoid 3′-hydroxylase (F3′H), flavonoid 3′,5′-hydroxylase (F3′5′H), dihydroflavonol 4-reductase (DFR), anthocyanidin synthase (ANS), flavonol synthase (FLS), and UDP-glucose flavonoid 3-O-glucosyltransferase (UFGT), were retrieved from Arabidopsis thaliana (TAIR) and other well-characterized plant species as queries [56]. Local BLASTP searches (E-value < 1 × 10^−5^) were performed against the sweetpotato protein database, followed by reciprocal BLAST against the Arabidopsis proteome to confirm orthology. Candidate proteins were further verified for the presence of characteristic functional domains using the Pfam (https://pfam.xfam.org/) and CDD databases (https://www.ncbi.nlm.nih.gov/Structure/cdd/cdd.shtml, 14 May 2026). Redundant isoforms were filtered based on the longest transcript per locus.

### 4.7. Transcriptome Sequencing Analysis of Genetically Transformed Plants

Wild-CK, *IbMYB11*-overexpressing (OE), and RNAi-silenced sweetpotato plants were subjected to simulated drought stress by treatment with 30% (*w*/*v*) PEG6000. In order to visually distinguish differences between the samples under normal conditions and those under PEG stress, all the sample IDs prefixed with “D” indicate pre-treated samples with PEG 6000. Leaf samples were collected at 0 h (CK) and 6 h post-treatment, with three independent biological replicates. Total RNA was extracted as described above, and its integrity and concentration were assessed using a NanoDrop spectrophotometer and an Agilent 2100 Bioanalyzer (Santa Clara, CA, USA, RNA integrity number ≥ 7.0). Strand-specific cDNA libraries were constructed using the TruSeq Stranded mRNA Library Prep Kit (Illumina, San Diego, CA, USA) according to the manufacturer’s instructions. The libraries were sequenced on the Illumina NovaSeq 6000 platform to generate 150 bp paired-end reads (Illumina, San Diego, CA, USA). Raw reads were filtered to remove adapter sequences and low-quality reads using Trimmomatic v0.39 [57]. Clean reads were aligned to the sweetpotato (*Ipomoea batatas*) reference genome (SweetGAINS I. batatasTanzania Phased v3, https://sweetpotato.uga.edu/) using HISAT2 v2.1.0 [58]. Gene expression levels were quantified as fragments per kilobase of transcript per million mapped reads (FPKM) using StringTie v2.1.4 [59]. Differentially expressed genes (DEGs) were identified using DESeq2 (v1.28.1) with the thresholds of |log2 fold change| ≥ 1 and adjusted *p* value (false discovery rate, FDR) < 0.05 [60]. Gene Ontology (GO) and Kyoto Encyclopedia of Genes and Genomes (KEGG) enrichment analyses of DEGs were performed using clusterProfiler v4.0 [61].

### 4.8. Metabolic Profiling Analysis of Genetically Transformed Plants

Half of each above drought-stressed leaf sample was extracted with 1.0 mL 70% methanol containing internal standards. The mixture was vortexed, ultrasonicated for 30 min, and centrifuged at 12,000× *g* for 10 min at 4 °C. The supernatant was filtered through a 0.22 μm polytetrafluoroethylene (PTFE) membrane and subjected to UPLC-MS/MS analysis.

Metabolite profiling was performed on a Waters Acquity I-Class PLUS UHPLC coupled with Waters Xevo G2-XS QTOF (Dynamics, Newcastle, UK) high-resolution mass spectrometer system by Adsen Biotech (Urumqi, China). Briefly, metabolite separation was performed on an ACQUITY UPLC HSS T3 column (1.8 μm, 2.1 × 100 mm) at 40 °C with a binary gradient of 0.1% formic acid in water (A) and acetonitrile (B): 0–1 min, 5% B; 1–9 min, 5–95% B; 9–10 min, 95% B; 10–10.1 min, 95–5% B; 10.1–12 min, 5% B. The flow rate was 0.3 mL min^−1^ and the injection volume was 2 μL. Mass spectrometric detection was conducted in both positive and negative electrospray ionization modes. Quality control (QC) samples, prepared by pooling aliquots of all sample extracts, were interspersed every 10 injections to monitor instrument stability. Raw data were processed using Progenesis QI v3.0 (Waters/Nonlinear Dynamics, Newcastle, UK) for peak picking, alignment, and annotation against metabolite databases (KEGG, HMDB, METLIN and an in-house library). Metabolite intensities were normalized to the total ion current. Multivariate analysis (PCA and OPLS-DA) was performed using SIMCA-P (v14.1). Differentially accumulated metabolites (DAMs) were identified with variable importance in projection (VIP) ≥ 1 derived from OPLS-DA, |log_2_ fold change| ≥ 1, and *p* < 0.05.

### 4.9. Combined WGCNA Analysis of Transcriptome and Flavonoid Metabolites

The reference genome’s TF was annotated by using PlantTF v5.0 (https://planttfdb.gao-lab.org/prediction.php, 4 June 2026). Differentially expressed TFs identified from the transcriptome dataset were selected as the input data set for WGCNA [62]. Expression matrices were log_2_-transformed and filtered to retain genes with a median absolute deviation (MAD) in the top 50% quantile. A soft-thresholding power (β) was chosen to achieve scale-free topology (R^2^ > 0.85). The topological overlap matrix (TOM) was computed and subjected to hierarchical clustering with the dynamic tree-cutting algorithm (deepSplit = 2, minModuleSize = 30). Modules with eigengene correlation > 0.75 were merged. Module eigengenes (MEs), defined as the first principal component of each module, were correlated with flavonoid metabolite accumulation profiles (log_2_-transformed normalized intensities) using Pearson correlation. Modules exhibiting the strongest correlation with flavonoid contents were designated as candidate modules, and hub genes were identified based on high module membership (kME > 0.8) and high gene significance (GS > 0.2).

### 4.10. Statistical Analysis

All physiological indicator experiments were performed with at least three biological replicates. Data are presented as mean ± standard error (SE) unless otherwise stated. For two-group comparisons, two-tailed Student’s *t*-tests were used. Significance levels are indicated as * *p* < 0.05 and ** *p* < 0.01.

## 5. Conclusions

This study identified *IbMYB11*, a nuclear-localized MYB transcription factor, as a positive regulator of drought tolerance in sweet potato. *IbMYB11* may enhance drought tolerance by increasing SOD and POD activities and promoting flavonoid biosynthesis, thereby strengthening the antioxidant capacity of transgenic plants. Integrated transcriptomic, metabolomic, and WGCNA analyses further revealed a transcriptional regulatory network connecting multiple TF families with flavonoid structural genes. These findings provide new insights into the molecular mechanisms underlying drought tolerance in sweet potato and offer valuable genetic resources for breeding drought-tolerant cultivars.

## Data Availability

Data supporting the findings of this work are available within the paper and the Supplementary Tables and Figures. The RNA-seq reads have been deposited into the China National Center for Bioinformation (https://www.cncb.ac.cn/; PRJCA070053).

## Supplementary Materials

The following supporting information can be downloaded at https://www.mdpi.com/article/10.3390/plants15203119/s1: Figure S1: Evolutionary tree of the MYB TFs; Figure S2: PCR-based gel electrophoresis detection of the *IbMYB11* transformed lines of sweet potatoes. (A). The gel electrophoresis patterns of the RNAi transformed plants (Ri-M) and the wild-type control. (B). The gel electrophoresis patterns of the Overexpression transformed plants (OE-M) and the wild-type control; Figure S3: Expression level of *IbMYB11* in *IbMYB11*-transgenic plants. (A) Relative expression levels of *IbMYB11* in *IbMYB11*-OE and CK plants. (B) Relative expression levels of *IbMYB11* in *IbMYB11*-RNAi and CK plants. For all RT-qPCR analyses, the control treatment was set to 1, and data are presented as mean ± SE of three biological replicates; Figure S4: Correlation of transcriptome expression levels of 18 samples; Figure S5: Box plot of transcriptome expression levels of 18 samples; Figure S6: Venn diagram of differentially expressed genes (DEGs) in different comparison groups. (A) DEGs in comparison groups of D*IbMYB11*-RNAi vs. DWild-CK, D*IbMYB11*-RNAi vs. Wild-CK and D*IbMYB11*-RNAi vs. *IbMYB11*-RNAi. (B) DEGs in comparison groups of D*IbMYB11*-OE vs. DWild-CK, D*IbMYB11*-OE vs. Wild-CK and D*IbMYB11*-OE vs. *IbMYB11*-OE. The column charts below indicate the number of DEGs in each comparison group; Figure S7: PCA analysis of the metabolic profiles of 18 samples for sample relationship evaluation and quality assessment; Figure S8: Volcano plot of differentially expressed metabolites (DEMs) in 6 comparison groups (D*IbMYB11*-RNAi vs. Wild-CK, D*IbMYB11*-RNAi vs. *IbMYB11*-OE, D*IbMYB11*-RNAi vs. DWild-CK, D*IbMYB11*-OE vs. Wild-CK, D*IbMYB11*-OE vs. *IbMYB11*-OE, and D*IbMYB11*-OE vs. DWild-CK). Table S1: Primers used in this paper; Table S2: A summary of RNAseq data and reference mapping statistics; Table S3: The hub transcription factors in each co-expression module; Table S4: Annotation of flavonoid pathway structural genes in the reference genome of sweet potato and differential expression analysis.
