# Supplementary material for: IbMYB11 Involved in Drought Stress Responses Through Association with Flavonoid Metabolism in Sweet Potato
Source: Plants (Basel). 2026 Oct 12;15(20):3119. doi: 10.3390/plants15203119 (PMC13709136; doi:10.3390/plants15203119)
Supplement: Supplementary file 1 [file plants-15-03119-s001.zip › Figure S1.pdf]

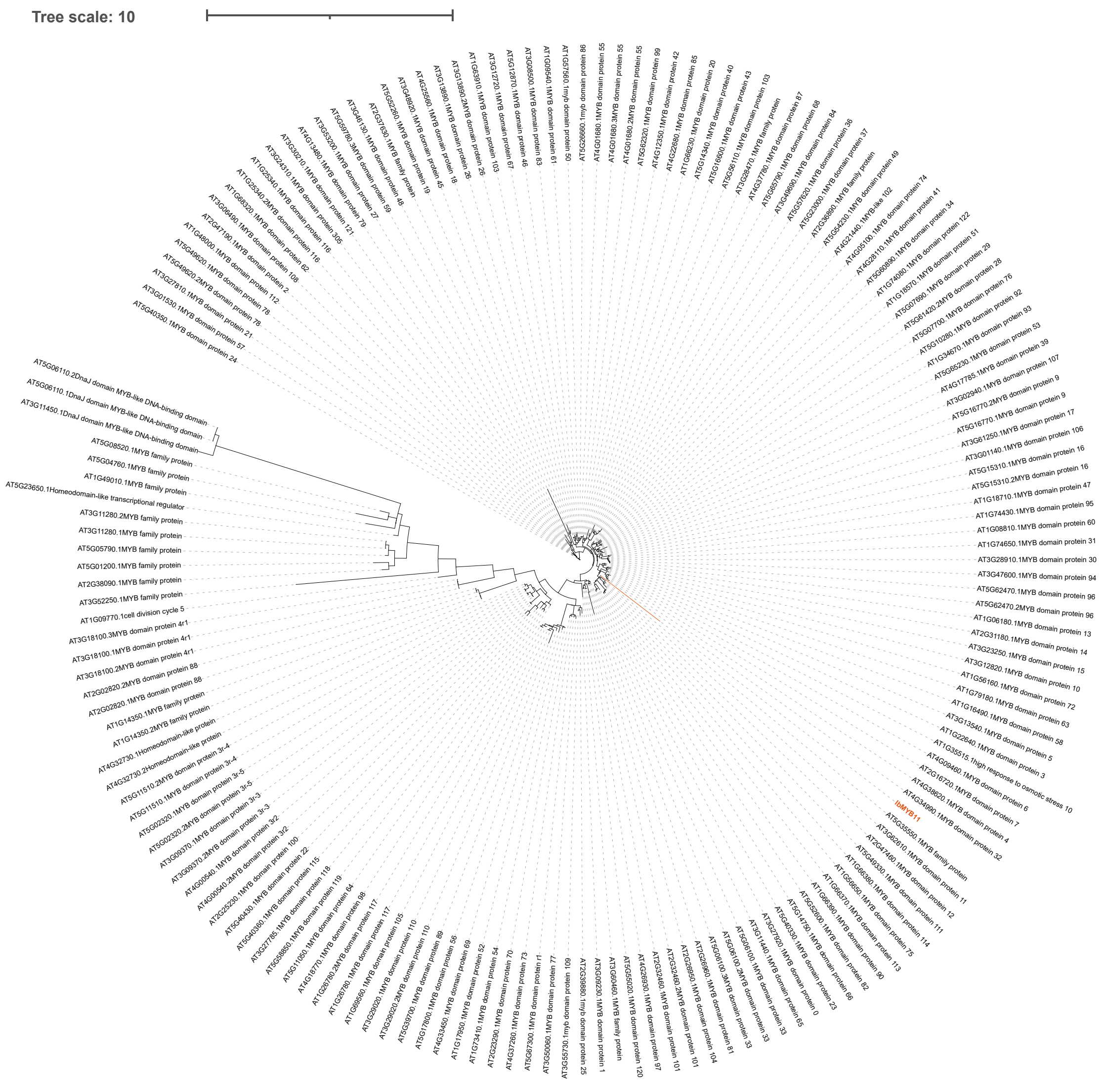
Tree scale: 10
AT5G06110.2DnaJ domain MYB-like DNA-binding domain
AT5G06110.1DnaJ domain MYB-like DNA-binding domain
AT3G11450.1DnaJ domain MYB-like DNA-binding domain
AT5G08520.1MYB family protein
AT5G04760.1MYB family protein
AT1G49010.1MYB family protein
AT5G23650.1Homeodomain-like transcriptional regulator
AT3G11280.2MYB family protein
AT3G11280.1MYB family protein
AT5G05790.1MYB family protein
AT5G01200.1MYB family protein
AT2G38090.1MYB family protein
AT3G52250.1MYB family protein
AT1G09770.1cell division cycle 5
AT3G18100.3MYB domain protein 4r1
AT3G18100.1MYB domain protein 4r1
AT3G18100.2MYB domain protein 4r1
AT2G02820.2MYB domain protein 88
AT2G02820.1MYB domain protein 88
AT1G14350.1MYB family protein
AT1G14350.2MYB family protein
AT4G32730.1Homeodomain-like protein
AT4G32730.2Homeodomain-like protein
AT5G11510.2MYB domain protein 3r-4
AT5G11510.1MYB domain protein 3r-4
AT5G02320.1MYB domain protein 3r-5
AT5G02320.2MYB domain protein 3r-5
AT3G09370.1MYB domain protein 3r-3
AT3G09370.2MYB domain protein 3r-3
AT4G00540.1MYB domain protein 3r2
AT4G00540.2MYB domain protein 3r2
AT2G25230.1MYB domain protein 100
AT5G40430.1MYB domain protein 22
AT5G40360.1MYB domain protein 115
AT3G27785.1MYB domain protein 118
AT5G58850.1MYB domain protein 119
AT5G11050.1MYB domain protein 64
AT4G18770.1MYB domain protein 98
AT1G26780.2MYB domain protein 117
AT1G26780.1MYB domain protein 117
AT1G69560.1MYB domain protein 105
AT3G29020.1MYB domain protein 110
AT3G29020.2MYB domain protein 110
AT5G39700.1MYB domain protein 89
AT5G17800.1MYB domain protein 56
AT4G33450.1MYB domain protein 69
AT1G17950.1MYB domain protein 52
AT1G73410.1MYB domain protein 54
AT2G23290.1MYB domain protein 70
AT4G37260.1MYB domain protein 73
AT5G67300.1MYB domain protein r1
AT3G50060.1MYB domain protein 77
AT3G55730.1myb domain protein 109
AT2G39880.1myb domain protein 25
AT3G09230.1MYB domain protein 1
AT3G60460.1MYB family protein
AT5G55020.1MYB domain protein 120
AT4G26930.1MYB domain protein 97
AT2G32460.1MYB domain protein 101
AT2G32460.2MYB domain protein 101
AT2G26950.1MYB domain protein 104
AT5G26960.1MYB domain protein 81
AT5G06100.3MYB domain protein 33
AT5G06100.2MYB domain protein 33
AT5G06100.1MYB domain protein 33
AT3G11440.1MYB domain protein 65
AT3G27920.1MYB domain protein 0
AT5G40330.1MYB domain protein 23
AT5G14750.1MYB domain protein 66
AT5G52600.1MYB domain protein 82
AT1G66390.1MYB domain protein 90
AT1G66370.1MYB domain protein 113
AT1G56650.1MYB domain protein 75
AT1G66380.1MYB domain protein 114
AT5G49330.1MYB domain protein 111
AT2G47460.1MYB domain protein 12
AT3G62610.1MYB domain protein 11
AT5G35550.1MYB family protein
IbMYB11
AT4G34990.1MYB domain protein 32
AT4G38620.1MYB domain protein 4
AT2G16720.1MYB domain protein 7
AT4G09460.1MYB domain protein 6
AT1G35515.1high response to osmotic stress 10
AT1G22640.1MYB domain protein 3
AT3G13540.1MYB domain protein 5
AT1G16490.1MYB domain protein 58
AT1G79180.1MYB domain protein 63
AT1G56160.1MYB domain protein 72
AT3G12820.1MYB domain protein 10
AT3G23250.1MYB domain protein 15
AT2G31180.1MYB domain protein 14
AT1G06180.1MYB domain protein 13
AT5G62470.2MYB domain protein 96
AT5G62470.1MYB domain protein 96
AT3G47600.1MYB domain protein 94
AT3G28910.1MYB domain protein 30
AT1G74650.1MYB domain protein 31
AT1G08810.1MYB domain protein 60
AT1G74430.1MYB domain protein 95
AT1G18710.1MYB domain protein 47
AT5G15310.2MYB domain protein 16
AT5G15310.1MYB domain protein 16
AT3G01140.1MYB domain protein 106
AT3G61250.1MYB domain protein 17
AT5G16770.1MYB domain protein 9
AT5G16770.2MYB domain protein 9
AT3G02940.1MYB domain protein 107
AT4G17785.1MYB domain protein 39
AT5G65230.1MYB domain protein 53
AT1G34670.1MYB domain protein 93
AT5G10280.1MYB domain protein 92
AT5G07700.1MYB domain protein 76
AT5G61420.2MYB domain protein 28
AT5G07690.1MYB domain protein 29
AT1G18570.1MYB domain protein 51
AT1G74080.1MYB domain protein 122
AT5G60890.1MYB domain protein 34
AT4G28110.1MYB domain protein 41
AT4G05100.1MYB domain protein 74
AT4G21440.1MYB-like 102
AT5G54230.1MYB domain protein 49
AT2G36890.1MYB family protein
AT5G23000.1MYB domain protein 37
AT5G57620.1MYB domain protein 36
AT3G49690.1MYB domain protein 84
AT5G65790.1MYB domain protein 68
AT4G37780.1MYB domain protein 87
AT3G28470.1MYB family protein
AT5G56110.1MYB domain protein 103
AT5G16600.1MYB domain protein 43
AT5G14340.1MYB domain protein 40
AT1G66230.1MYB domain protein 20
AT4G22680.1MYB domain protein 85
AT4G12350.1MYB domain protein 42
AT5G62320.1MYB domain protein 99
AT4G01680.2MYB domain protein 55
AT4G01680.3MYB domain protein 55
AT4G01680.1MYB domain protein 55
AT5G26660.1myb domain protein 86
AT1G57560.1myb domain protein 50
AT1G09540.1MYB domain protein 61
AT3G08500.1MYB domain protein 83
AT5G12870.1MYB domain protein 46
AT3G12720.1MYB domain protein 67
AT1G63910.1MYB domain protein 103
AT3G13890.2MYB domain protein 26
AT3G13890.1MYB domain protein 26
AT4G25560.1MYB domain protein 18
AT3G48920.1MYB domain protein 45
AT5G52260.1MYB domain protein 19
AT2G37630.1MYB family protein
AT3G46130.1MYB domain protein 48
AT5G59780.3MYB domain protein 59
AT3G53200.1MYB domain protein 27
AT4G13480.1MYB domain protein 79
AT3G30210.1MYB domain protein 121
AT3G24310.1MYB domain protein 305
AT1G25340.1MYB domain protein 116
AT1G25340.2MYB domain protein 116
AT1G68320.1MYB domain protein 62
AT3G06490.1MYB domain protein 108
AT2G47190.1MYB domain protein 2
AT1G48000.1MYB domain protein 112
AT5G49620.1MYB domain protein 78
AT5G49620.2MYB domain protein 78
AT3G27810.1MYB domain protein 21
AT3G01530.1MYB domain protein 57
AT5G40350.1MYB domain protein 24
