# Supplementary material for: IbMYB11 Involved in Drought Stress Responses Through Association with Flavonoid Metabolism in Sweet Potato
Source: Plants (Basel). 2026 Oct 12;15(20):3119. doi: 10.3390/plants15203119 (PMC13709136; doi:10.3390/plants15203119)
Supplement: Supplementary file 1 [file plants-15-03119-s001.zip › Figure S2.pdf]

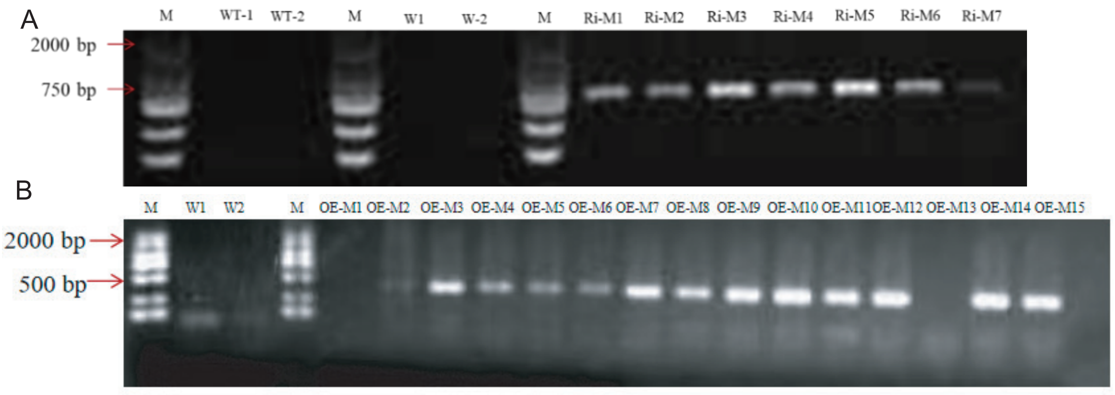
A
B
M
WT-1
WT-2
M
W1
W-2
M
Ri-M1
Ri-M2
Ri-M3
Ri-M4
Ri-M5
Ri-M6
Ri-M7
2000 bp
750 bp
M
W1
W2
M
OE-M1
OE-M2
OE-M3
OE-M4
OE-M5
OE-M6
OE-M7
OE-M8
OE-M9
OE-M10
OE-M11
OE-M12
OE-M13
OE-M14
OE-M15
2000 bp
500 bp
