# Supplementary material for: IbMYB11 Involved in Drought Stress Responses Through Association with Flavonoid Metabolism in Sweet Potato
Source: Plants (Basel). 2026 Oct 12;15(20):3119. doi: 10.3390/plants15203119 (PMC13709136; doi:10.3390/plants15203119)
Supplement: Supplementary file 1 [file plants-15-03119-s001.zip › Figure S3.pdf]

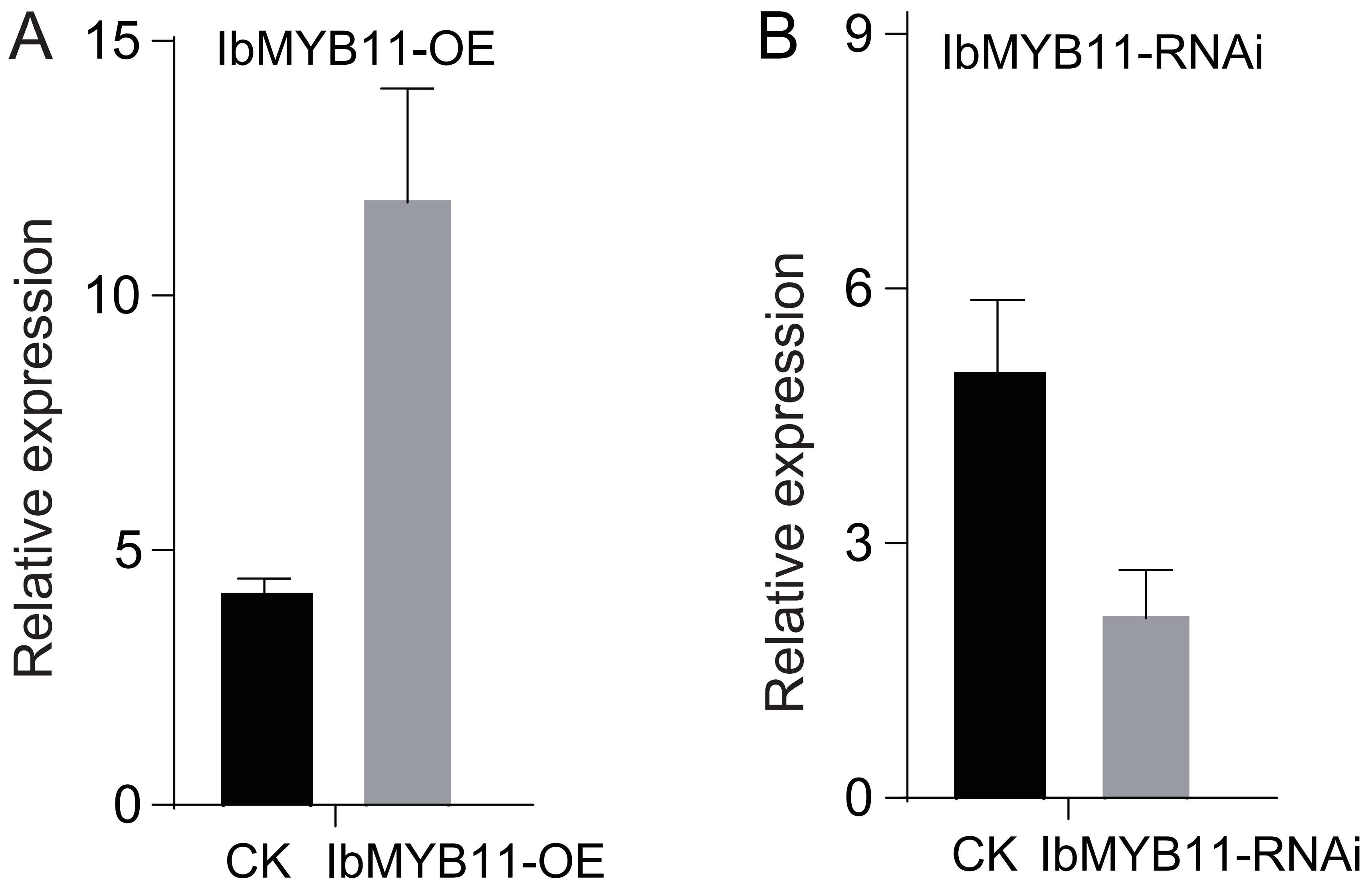
A
IbMYB11-OE
15
10
5
0
Relative expression
CK
IbMYB11-OE
B
IbMYB11-RNAi
9
6
3
0
Relative expression
CK
IbMYB11-RNAi
