# Supplementary material for: IbMYB11 Involved in Drought Stress Responses Through Association with Flavonoid Metabolism in Sweet Potato
Source: Plants (Basel). 2026 Oct 12;15(20):3119. doi: 10.3390/plants15203119 (PMC13709136; doi:10.3390/plants15203119)
Supplement: Supplementary file 1 [file plants-15-03119-s001.zip › Figure S4.pdf]

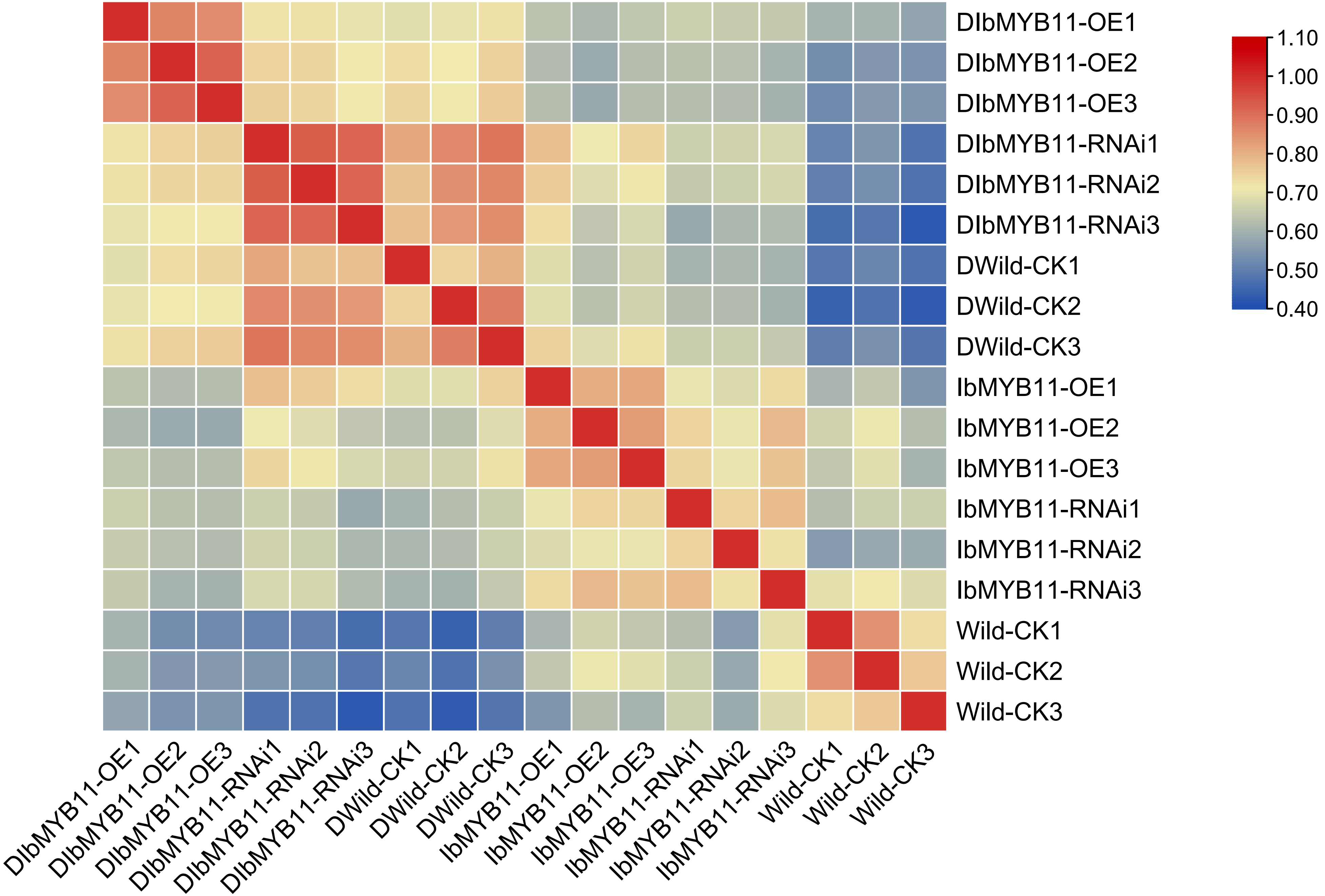
DIbMYB11-OE1
DIbMYB11-OE2
DIbMYB11-OE3
DIbMYB11-RNAi1
DIbMYB11-RNAi2
DIbMYB11-RNAi3
DWild-CK1
DWild-CK2
DWild-CK3
IbMYB11-OE1
IbMYB11-OE2
IbMYB11-OE3
IbMYB11-RNAi1
IbMYB11-RNAi2
IbMYB11-RNAi3
Wild-CK1
Wild-CK2
Wild-CK3
1.10
1.00
0.90
0.80
0.70
0.60
0.50
0.40
