# Supplementary material for: IbMYB11 Involved in Drought Stress Responses Through Association with Flavonoid Metabolism in Sweet Potato
Source: Plants (Basel). 2026 Oct 12;15(20):3119. doi: 10.3390/plants15203119 (PMC13709136; doi:10.3390/plants15203119)
Supplement: Supplementary file 1 [file plants-15-03119-s001.zip › Figure S5.pdf]

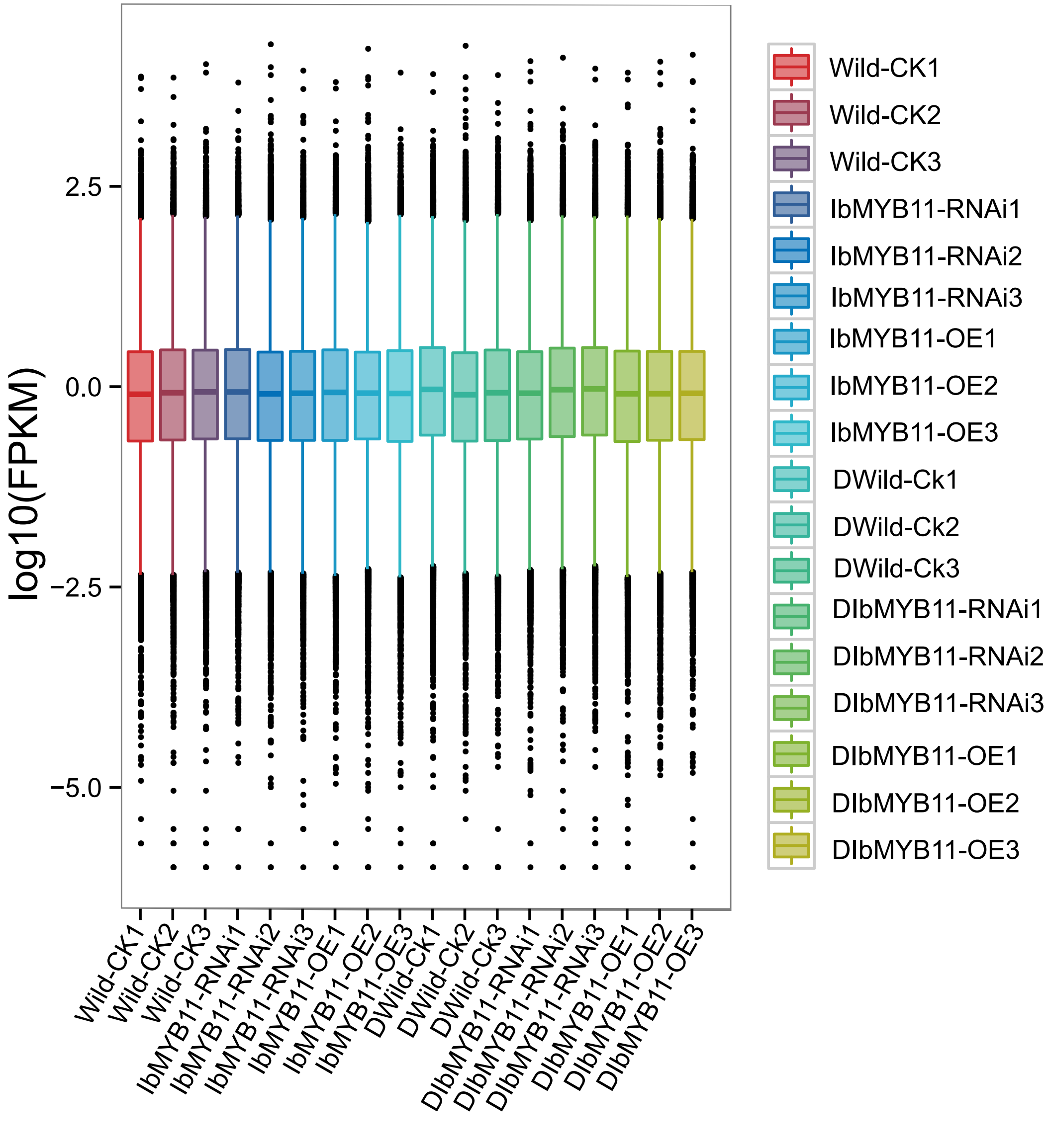

log10(FPKM)
2.5
0.0
−2.5
−5.0
Wild-CK1
Wild-CK2
Wild-CK3
IbMYB11-RNAi1
IbMYB11-RNAi2
IbMYB11-RNAi3
IbMYB11-OE1
IbMYB11-OE2
IbMYB11-OE3
DWild-Ck1
DWild-Ck2
DWild-Ck3
DIbMYB11-RNAi1
DIbMYB11-RNAi2
DIbMYB11-RNAi3
DIbMYB11-OE1
DIbMYB11-OE2
DIbMYB11-OE3
