# Supplementary material for: IbMYB11 Involved in Drought Stress Responses Through Association with Flavonoid Metabolism in Sweet Potato
Source: Plants (Basel). 2026 Oct 12;15(20):3119. doi: 10.3390/plants15203119 (PMC13709136; doi:10.3390/plants15203119)
Supplement: Supplementary file 1 [file plants-15-03119-s001.zip › Figure S6.pdf]

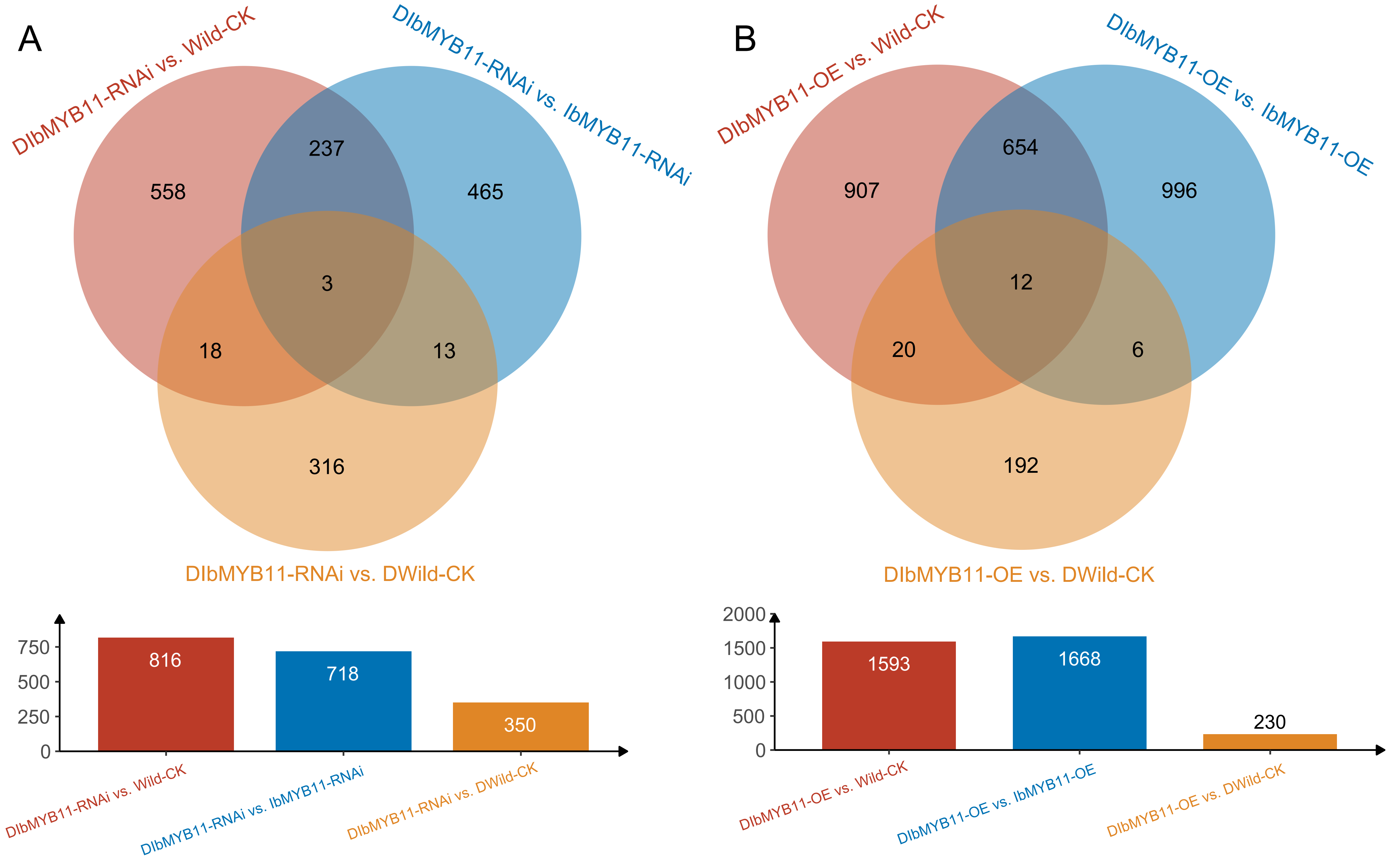
A
DIbMYB11-RNAi vs. Wild-CK
DIbMYB11-RNAi vs. IbMYB11-RNAi
558
237
465
3
18
13
316
DIbMYB11-RNAi vs. DWild-CK
750
500
250
0
816
718
350
DIbMYB11-RNAi vs. Wild-CK
DIbMYB11-RNAi vs. IbMYB11-RNAi
DIbMYB11-RNAi vs. DWild-CK
B
DIbMYB11-OE vs. Wild-CK
DIbMYB11-OE vs. IbMYB11-OE
907
654
996
12
20
6
192
DIbMYB11-OE vs. DWild-CK
2000
1500
1000
500
0
1593
1668
230
DIbMYB11-OE vs. Wild-CK
DIbMYB11-OE vs. IbMYB11-OE
DIbMYB11-OE vs. DWild-CK
