# Supplementary material for: IbMYB11 Involved in Drought Stress Responses Through Association with Flavonoid Metabolism in Sweet Potato
Source: Plants (Basel). 2026 Oct 12;15(20):3119. doi: 10.3390/plants15203119 (PMC13709136; doi:10.3390/plants15203119)
Supplement: Supplementary file 1 [file plants-15-03119-s001.zip › Figure S7.pdf]

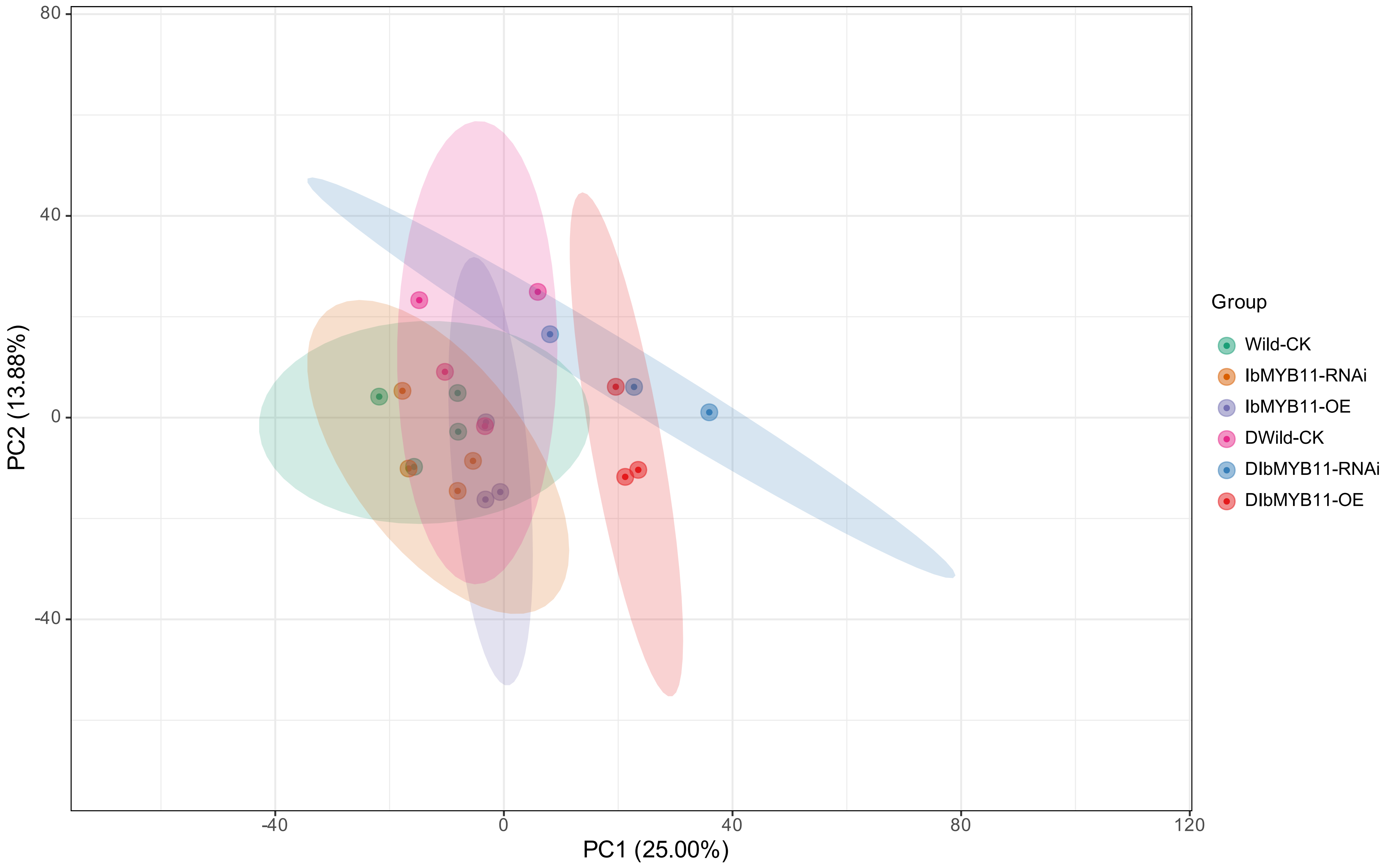

80
40
0
-40
PC2 (13.88%)
-40
0
40
80
120
PC1 (25.00%)
Group
Wild-CK
IbMYB11-RNAi
IbMYB11-OE
DWild-CK
DIbMYB11-RNAi
DIbMYB11-OE
