# Supplementary material for: IbMYB11 Involved in Drought Stress Responses Through Association with Flavonoid Metabolism in Sweet Potato
Source: Plants (Basel). 2026 Oct 12;15(20):3119. doi: 10.3390/plants15203119 (PMC13709136; doi:10.3390/plants15203119)
Supplement: Supplementary file 1 [file plants-15-03119-s001.zip › Figure S8.pdf]

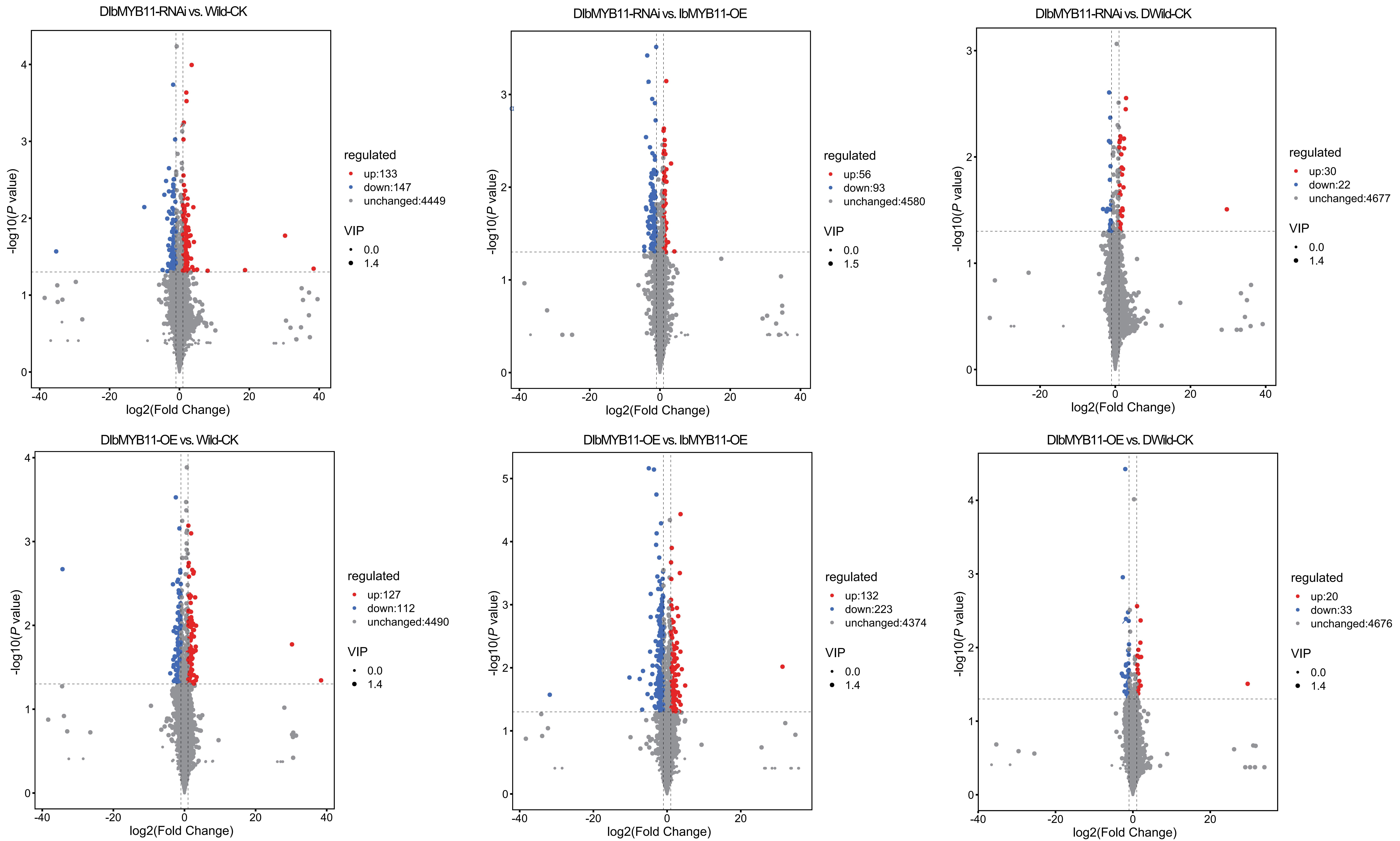
DIbMYB11-RNAi vs. Wild-CK
regulated
up:133
down:147
unchanged:4449
VIP
0.0
1.4
DIbMYB11-RNAi vs. IbMYB11-OE
regulated
up:56
down:93
unchanged:4580
VIP
0.0
1.5
DIbMYB11-RNAi vs. DWild-CK
regulated
up:30
down:22
unchanged:4677
VIP
0.0
1.4
DIbMYB11-OE vs. Wild-CK
regulated
up:127
down:112
unchanged:4490
VIP
0.0
1.4
DIbMYB11-OE vs. IbMYB11-OE
regulated
up:132
down:223
unchanged:4374
VIP
0.0
1.4
DIbMYB11-OE vs. DWild-CK
regulated
up:20
down:33
unchanged:4676
VIP
0.0
1.4
-log10(P value)
log2(Fold Change)
